# Liver cirrhosis mortality in 187 countries between 1980 and 2010: a systematic analysis

**DOI:** 10.1186/s12916-014-0145-y

**Published:** 2014-09-18

**Authors:** Ali A Mokdad, Alan D Lopez, Saied Shahraz, Rafael Lozano, Ali H Mokdad, Jeff Stanaway, Christopher JL Murray, Mohsen Naghavi

**Affiliations:** Institute for Health Metrics and Evaluation, University of Washington, Seattle, WA USA; Department of Surgery, University of Texas Southwestern, Dallas, TX USA; School of Population and Global Health, University of Melbourne, Carlton, VIC Australia; Schneider Institute for Health Policy, Brandeis University, Waltham, MA USA

**Keywords:** Liver cirrhosis, Mortality, Global, Alcohol, Hepatitis

## Abstract

**Background:**

Liver cirrhosis is a major yet largely preventable and underappreciated cause of global health loss. Variations in cirrhosis mortality at the country level reflect differences in prevalence of risk factors such as alcohol use and hepatitis B and C infection. We estimated annual age-specific mortality from liver cirrhosis in 187 countries between 1980 and 2010.

**Methods:**

We systematically collected vital registration and verbal autopsy data on liver cirrhosis mortality for the period 1980 to 2010. We corrected for misclassification of deaths, which included deaths attributed to improbable or nonfatal causes. We used ensemble models to estimate liver cirrhosis mortality with uncertainty by age, sex, country and year. We used out-of-sample predictive validity to select the optimal model.

**Results:**

Global liver cirrhosis deaths increased from around 676,000 (95% uncertainty interval: 452,863 to 1,004,530) in 1980 to over 1 million (1,029,042; 670,216 to 1,554,530) in 2010 (about 2% of the global total). Over the same period, the age-standardized cirrhosis mortality rate decreased by 22%. This was largely driven by decreasing cirrhosis mortality rates in China, the US and countries in Western Europe. In 2010, Egypt, followed by Moldova, had the highest age-standardized cirrhosis mortality rates, 72.7 and 71.2 deaths per 100,000, respectively, while Iceland had the lowest. In Egypt, almost one-fifth (18.1%) of all deaths in males 45- to 54-years old were due to liver cirrhosis. Liver cirrhosis mortality in Mexico is the highest in Latin America. In France and Italy, liver cirrhosis mortality fell by 50% to 60%; conversely, in the United Kingdom, mortality increased by about one-third. Mortality from liver cirrhosis was also comparatively high in Central Asia countries, particularly Mongolia, Uzbekistan and Kyrgyzstan, and in parts of sub-Saharan Africa, notably Gabon.

**Conclusions:**

Liver cirrhosis is a significant cause of global health burden, with more than one million deaths in 2010. Our study identifies areas with high and/or rapidly increasing mortality where preventive measures to control and reduce liver cirrhosis risk factors should be urgently strengthened.

Please see related commentary: http://www.biomedcentral.com/1741-7015/12/159/abstract.

**Electronic supplementary material:**

The online version of this article (doi:10.1186/s12916-014-0145-y) contains supplementary material, which is available to authorized users.

## Background

Liver cirrhosis has emerged as a major cause of global health burden. According to the Global Burden of Disease 2010 study, liver cirrhosis caused 31 million Disability Adjusted Life Years (DALYs), or 1.2% of global DALYs, in 2010, and one million deaths, or 2% of all deaths worldwide in that year [1,2]. The epidemiology of liver cirrhosis has been evaluated extensively in several developed countries in Europe and the Americas. There has been much less interest in mortality from the disease, however, in much of the developing world [3–6], in part because of poor data. The global health community has increasingly recognized the importance of controlling liver cirrhosis risk factors, particularly heavy alcohol intake and chronic viral hepatitis B and C infections. In 2005, the World Health Organization (WHO) Western Pacific Region adopted a regional goal to control hepatitis B by the year 2012 [7]. Earlier, the World Health Assembly in 1992 had agreed to integrate hepatitis B virus vaccine into national immunization programs [8]. In 2010, the Hepatitis B and C Summit Conference marked another step toward the control of the escalating impact of hepatitis B and C in Europe [9]. Known detrimental effects of alcohol consumption and its strong association with liver cirrhosis mortality have led to national and international policy responses to curtail alcohol consumption. Most recently, WHO hosted a high-level meeting to accelerate the implementation of a global strategy to reduce the harmful effects of increased alcohol consumption [10].

Since the original Global Burden of Disease study in 1990 [11], there has been no comprehensive global assessment of liver cirrhosis mortality. This is now possible, and timely, due to advances made by the Global Burden of Disease 2010 study (GBD 2010). First, an extensive database on mortality by cause for the years 1980 to 2010 has been collated, critically appraised and synthesized [2,12–14]. Second, advanced statistical methods for cause of death estimation, particularly in populations with incomplete vital registration and certification of deaths, have been developed [15]. Third, major advances with low-cost and high computational power computer equipment have facilitated objective assessments of model performance with out-of-sample predictive validity. Fourth, as part of the GBD 2010 process, a community of experts in the epidemiology of the disease has critically appraised the methods and results. In this paper, we present the key findings on liver cirrhosis mortality from the GBD 2010 study. For the first time, we provide estimates of annual age- and sex-specific liver cirrhosis mortality rates, with uncertainty, for 187 countries between 1980 and 2010.

## Methods

### Overview

Our aim was to estimate annual liver cirrhosis mortality levels, patterns, and temporal trends between 1980 and 2010 for 187 countries covering 99.7% of the global population. An empirical database on mortality due to liver cirrhosis was collated using primarily vital registration data. The data were systematically processed to enhance quality and comparability. We tested several models and assessed their performance using out-of-sample predictive validity. A more detailed description of our database synthesis and mortality estimation methods can be found in the published literature elsewhere [2,13–18] and is summarized below. The Institutional Review Board of the University of Washington and the Institute for Health Metrics and Evaluation approved the conduct of this study.

### Case definition of liver cirrhosis

The International Classification of Diseases (ICD) instruction manual permits assignment of an ICD code to the underlying cause of death as noted in a death certificate [19]. Depending on information available on the death certificate, a specific cause of death is assigned, for example, alcoholic cirrhosis of the liver, or a more general code can only be assigned, for example, unspecified cirrhosis of the liver. The immediate and intermediate causes of death are attributed to the underlying cause. Consequently, there has been large variation in assignment of ICD codes to liver cirrhosis deaths across the published literature, and no standard definition has been consistently utilized [20–23]. Based on the GBD2010 Gastrointestinal Diseases expert group recommendations, we included in our definition the following underlying causes of death: liver cirrhosis, chronic viral hepatitis infections and hepatic decompensation events. Table 1 expands on the specific ICD codes used and Additional file 1: Table S1 specifies the fraction of liver cirrhosis deaths under each ICD code. We opted for a broad definition in order to better accommodate differences across ICD revisions, variation in diagnostic accuracy across countries and discrepancies in reporting causes of death in different cultural contexts; for example, purposefully under-reporting deaths due to alcohol-related liver cirrhosis in cultures that prohibit alcohol intake. Deaths due to hepatocellular carcinoma were excluded. While hepatocellular carcinoma generally develops on a background of liver cirrhosis, we elected to keep deaths due to this malignant entity separate for several reasons: 1) ICD rules on coding causes of death assign hepatocellular carcinoma as an underlying cause of death irrespective of the presence or absence of liver cirrhosis [19]; 2) the distinction between liver cirrhosis and hepatocellular carcinoma is possible with minimal misclassification in countries with adequate cause of death data; and 3) the implication on survival and the management of each condition are different.

**Table 1:** **GBD cause mapping for the International Classification of Diseases**

**GBD name**	**ICD 10**	**ICD 9**	**ICD 9 BTL**
**Cirrhosis of the liver**	B18, I85, K70, K71.7, K72.1-K72.9, K73, K74, K75.2‐K75.9, K76.6‐K76.7, K76.9	070.22, 070.23, 070.32, 070.33, 070.44-070.49, 070.54‐070.59, 456.0‐456.2,571, 572.3‐572.8, 573	347

GBD, Global Burden of Disease, ICD, International Classification of Diseases. BTL = Basic Tabulation List.

### Data collection and processing

We first sought to collate all available vital registration records from 1980 to 2010. In total, we were able to identify cirrhosis deaths from 2,667 country-years from the vital registration data. We complemented this data collection with 80 site-years of published and unpublished verbal autopsy information. Verbal autopsy is a means for determining the cause of death in countries with incomplete or absent vital registration. It consists of a standardized questionnaire administered by a trained interviewer to a relative(s) of the deceased. The data gathered are then reviewed by a physician, matched against a predefined expert algorithm, or entered into a statistical model in order to assign a cause of death [24]. In the case of liver cirrhosis, data were predominantly certified by a physician. Admittedly, verbal autopsy data are highly heterogeneous owning to multiple questionnaire versions, different recall periods, variable age and sex groups, distinct cultures and variable methods for assigning cause of deaths. We did not correct for variability in verbal autopsy methods; nevertheless, we scrutinized the data for specific inclusion criteria: 1) sample size >50 and 2) methods compatible with standard verbal autopsy methods. There were no data available from Central Sub-Saharan Africa and verbal autopsy comprised the majority of information accrued for Eastern and Western Sub-Saharan Africa. Table 2 summarizes the number of site-years for each GBD region by decade and data source type. A complete list of all data sources is provided in Additional file 2: Table S2. Next, a specific set of ICD codes, those listed in Table 1, was mapped to liver cirrhosis in an attempt to enhance comparability across the different ICD revisions and variants. Another subset of ICD codes deemed ‘garbage codes’ was also redistributed onto liver cirrhosis. Garbage codes are codes of implausible, inappropriate or non-specific causes of death that were identified as underlying causes of death on death certificates. Examples of garbage codes redistributed onto liver cirrhosis include hematemesis and unspecified iseases of the digestive system. The redistribution process described by Naghavi *et al*. [13] reallocates a fraction of deaths assigned a garbage code to a probable target code, here liver cirrhosis, using methods such as proportional redistribution within an age-sex group, statistical models, and/or expert judgment. Liver cirrhosis deaths increased by 26% after redistribution of deaths assigned garbage codes; 40% of those were ill-defined GI signs and symptoms. Additional file 3: Table S3 lists all garbage codes redistributed onto liver cirrhosis and specifies the fraction redistributed and the consequent percent increase in liver cirrhosis deaths. Last, we systematically screened the data for outliers. Of 98,445 age- and sex-specific observations on cirrhosis deaths, 1,807 (or 1.8%) were identified as outliers based on the following criteria: implausible cause fractions, major inconsistencies with other sources within the same country, or pronounced deviations in mortality rates from comparator countries.

**Table 2:** **Site‐years by decade and source type in the mortality for each GBD region**

	**1980 to 1989**		**1990 to 1999**		**2000 to 2010**	
**Region**	**VA**	**VR**	**VA**	**VR**	**VA**	**VR**
**Asia Pacific, High Income**	0	25	0	34	0	33
**Asia, Central**	0	56	0	79	0	51
**Asia, East**	0	13	0	21	1	18
**Asia, South**	20	0	13	10	11	2
**Asia, Southeast**	0	46	0	37	7	52
**Australasia**	0	20	0	20	0	16
**Caribbean**	0	127	0	135	0	147
**Europe, Central**	0	62	0	97	0	115
**Europe, Eastern**	0	55	0	69	0	70
**Europe, Western**	0	200	0	201	0	204
**Latin America, Andean**	0	18	0	19	0	19
**Latin America, Central**	0	69	0	71	0	78
**Latin America, South**	0	30		29	0	28
**Latin America, Tropical**	0	20	0	18	0	21
**North Africa/Middle East**	0	26	0	26	1	82
**North America, High Income**	0	20	0	20	0	20
**Oceania**	0	1	0	10	0	4
**Sub-Saharan Africa, Central**	0	0	0	0	0	0
**Sub-Saharan Africa, East**	0	0	6	0	13	0
**Sub-Saharan Africa, Southern**	0	0	0	9	3	10
**Sub-Saharan Africa, West**	0	2	1	0	4	2
**Total**	**20**	**790**	**20**	**905**	**40**	**972**

GBD, Global Burden of Disease; VA, verbal autopsy; VR, vital registration.

### Model development

We estimated mortality levels and uncertainty intervals using cause of death ensemble modeling (CODEm) developed for cause of death estimation for the GBD 2010 study. A more detailed explanation of CODEm can be found elsewhere [15]. In brief, a large number of statistical models are created using combinations of selected covariates independently associated with the disease based on the literature. Plausible models then compete and are assessed by means of out-of-sample performance metrics.

We tested the following time series covariates: population-level alcohol consumption, health system access, prevalence of chronic hepatitis B and C infections, schistosomiasis, diabetes mellitus, body mass index (BMI), education and income. Population-level alcohol consumption estimates were constructed using annual national data on domestic production of alcoholic and fermented beverages, wine and beer. This was derived primarily from the national food balance sheets produced by the UN Food and Agriculture Organization (FAO) [25]. Health system access is a composite covariate, approximated from a principal component analysis of antenatal clinics, diphtheria-tetanus-pertussis immunization, measles immunization, in‐facility delivery and skilled birth attendance. Prevalence of chronic hepatitis B and C infections was estimated from data on hepatitis B surface antigen (HBsAg) and anti-hepatitis C virus (anti-HCV) antibody serology tests, respectively. We estimated country-level prevalence of chronic hepatitis viral infection for all years between 1980 and 2010 using DisMod 3, a meta-regression tool described in more detail elsewhere [1,26]. Schistosomiasis prevalence was derived from published global schistosomiasis atlases [27–29]. We estimated age-standardized diabetes prevalence using meta-regression in DisMod 3 from data on fasting plasma glucose, postprandial blood glucose and hemoglobin A1c.

We modeled all possible combinations of covariates and retained those combinations where the sign on the coefficients was in the expected direction, based on the literature, and if the coefficients were statistically significant (*P*-value <0.05). For each retained covariate combination, we developed four statistical models: (1) mixed effects linear models of the log of the death rate; (2) mixed effects linear models of the logit of the cause fraction; (3) spatial-temporal Gaussian process regression (ST-GPR) models of the log of the death rate; and (4) ST-GPR of the logit of the cause fraction [15]. This approach generated 474 models, 248 for males and 226 for females. Based on out-of-sample predictive performance of each individual model, we constructed ensemble models or blends of these various individual models. We assessed predictive validity of the ensemble and all component individual models using two out-of-sample performance metrics. First, we evaluated the predictive ability of every model using the root mean squared error (RMSE) of the logarithm of the death rate. Second, we assessed the percentage of time the model correctly predicted the temporal trend via a trend test. Based on the sum of ranks in these two metrics, the best performing component model or ensemble was selected. Additional file 4: Table S4 shows the optimal sex-specific ensemble models, the weight contributed by each submodel and the covariates included in each submodel.

**Figure 1:**
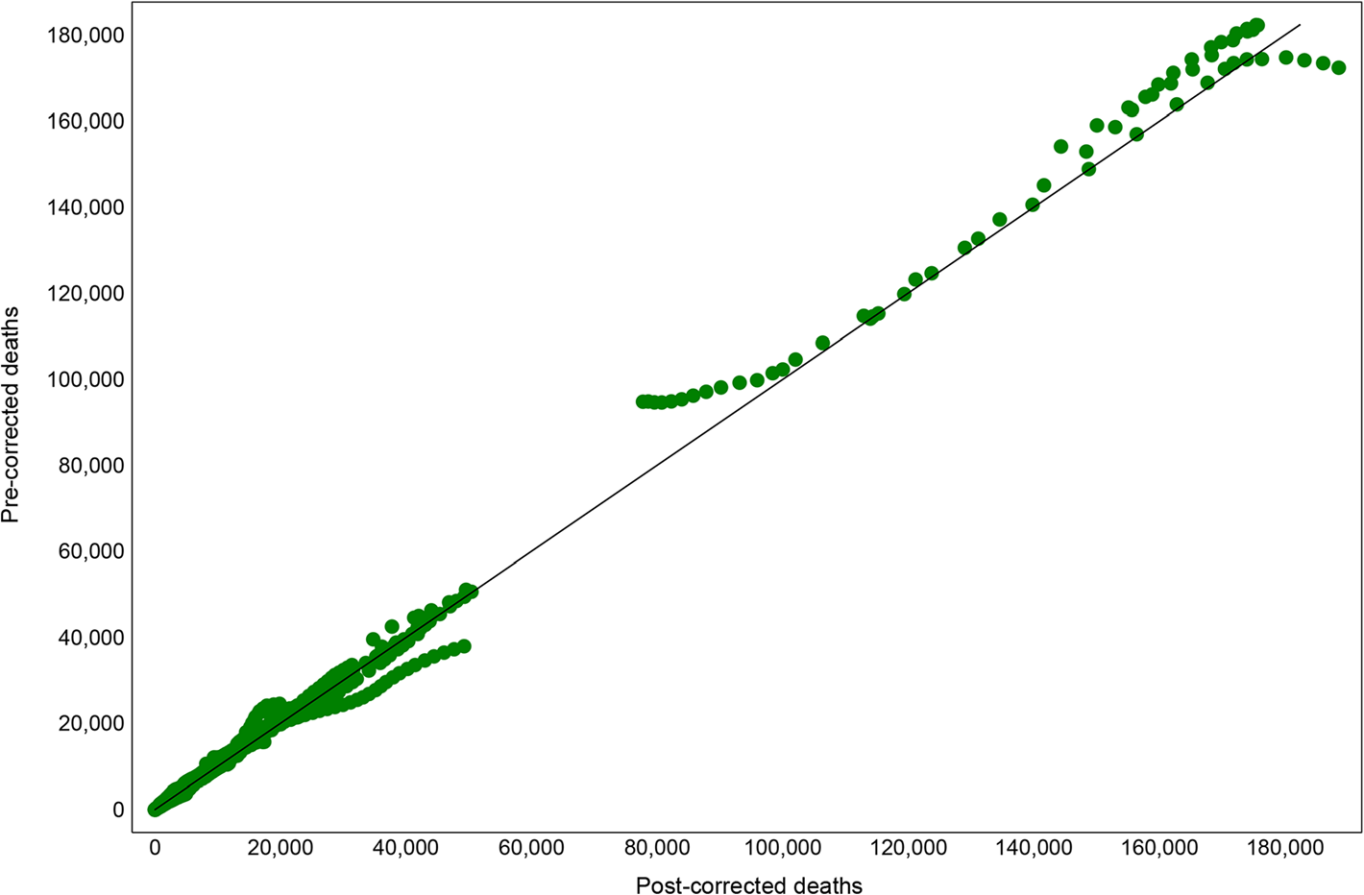
**Pre-corrected versus post-corrected liver cirrhosis deaths.**

The final step in estimating causes of death was to rescale the predicted deaths for each cause in the GBD study to fit the overall mortality envelope for each age-sex-year-country group, obtained separately from demographic analyses [12]. In other words, we constrain the sum of cause‐specific mortality rates, estimated in an unconstrained environment, to equal the mortality rate from all causes. Each cause was rescaled according to the uncertainty around its mortality estimate; causes that are known with relative precision were affected less by rescaling than causes that had large uncertainty. Figure 1 compares liver cirrhosis deaths before and after rescaling. Lozano *et al*. provides a more detailed explanation of this correction algorithm [2].

### Ancillary analyses

#### Decomposition of number of deaths into demographic and epidemiological factors

We decomposed the change in the number of liver cirrhosis deaths between 1990 and 2010 to help elucidate drivers of change. We computed the number of deaths expected in 2010 assuming two counterfactual scenarios: 1) a population growth scenario where population change between 1990 and 2010 is incorporated but the age-sex structure and liver cirrhosis mortality rates remain the same as in 1990; and 2) a population growth and aging scenario using 1990 age-sex specific rates and 2010 age-sex population numbers. We subsequently calculated the change in numbers of deaths related to population growth, population aging, and change in age-sex specific rates by observing the difference between the 1990 deaths and the population growth scenario, the population growth scenario and the population growth and aging scenario, and the 2010 deaths and population growth and aging scenario, respectively. A more detailed description of these methods can be found in Lozano *et al*. [2] Results at country-level are summarized in Additional file 5: Table S5.

#### Population attributable fractions for liver cirrhosis

We estimated population attributable fractions (PAFs) for liver cirrhosis related to heavy alcohol consumption, hepatitis B virus, hepatitis C virus and a residual category of unexplained risks denoted ‘other’. We estimated this component as follows: First, we reviewed the published literature on prevalence of the aforementioned exposures among confirmed liver cirrhosis cases. We defined chronic and heavy exposure to alcohol as any case with reported alcoholism, alcohol abuse, history of alcoholic liver disease or alcohol intake exceeding 20 g/day for at least five years, when specified. Chronic infection with hepatitis B and hepatitis C were defined as a positive HbsAg test and anti-HCV serology, respectively. Those cases that were not attributable to chronic alcohol intake and who tested negative for HBsAg and anti-HCV antibodies were defined as ‘other’. We identified 31, 55, 74 and 26 studies on the prevalence of alcoholism, hepatitis B infection, hepatitis C infection, and others in patients with liver cirrhosis, respectively. The data derived from 43 developed and developing countries and encompassed 19 GBD regions and the period between 1988 and 2009. Next, we estimated country- and year-specific exposure fractions among liver cirrhosis cases using meta-regression in DisMod 3. DisMod3 is a statistical software that employs an age-integrating mixed-effects negative-binomial model of relevant epidemiological data. We modeled each exposure separately and adjusted for alcohol intake, hepatitis B infection and hepatitis C infection. Then, we rescaled the fractions of exposure in cases to sum to one according to the uncertainty around each fraction as calculated in the meta-regression. We used the resulting predicted exposure fractions among prevalent cases as a proxy for PAFs for liver cirrhosis. This follows from the direct method of approximating PAFs:

$$ PA{F}_{e,c}={P}_{e,c}\times \frac{RP-1}{RR} $$

where

P_e,c_ approximates prevalence of exposure, e, in case, c

RR approximates relative risk of acquiring disease, c, given exposure e

**Figure 2:**
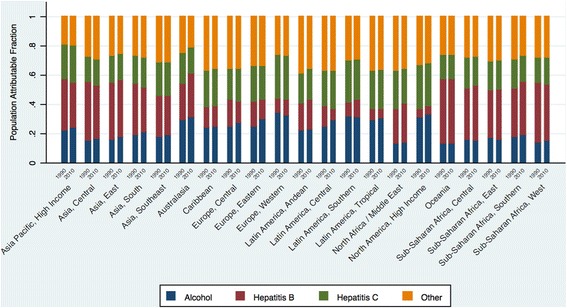
**Population attributable fractions in liver cirrhosis by region in 1990 and 2010.**

When the RR estimate is high, as is the case here, one can approximate P_e,c_ to the PAF estimate. A more detailed description of these methods can be found in the following selected references [26,30–35]. Figure 2 and Additional file 6: Table S6 depict PAF estimates for each exposure type by GBD region and for the years 1990 and 2010.

## Results

**Figure 3:**
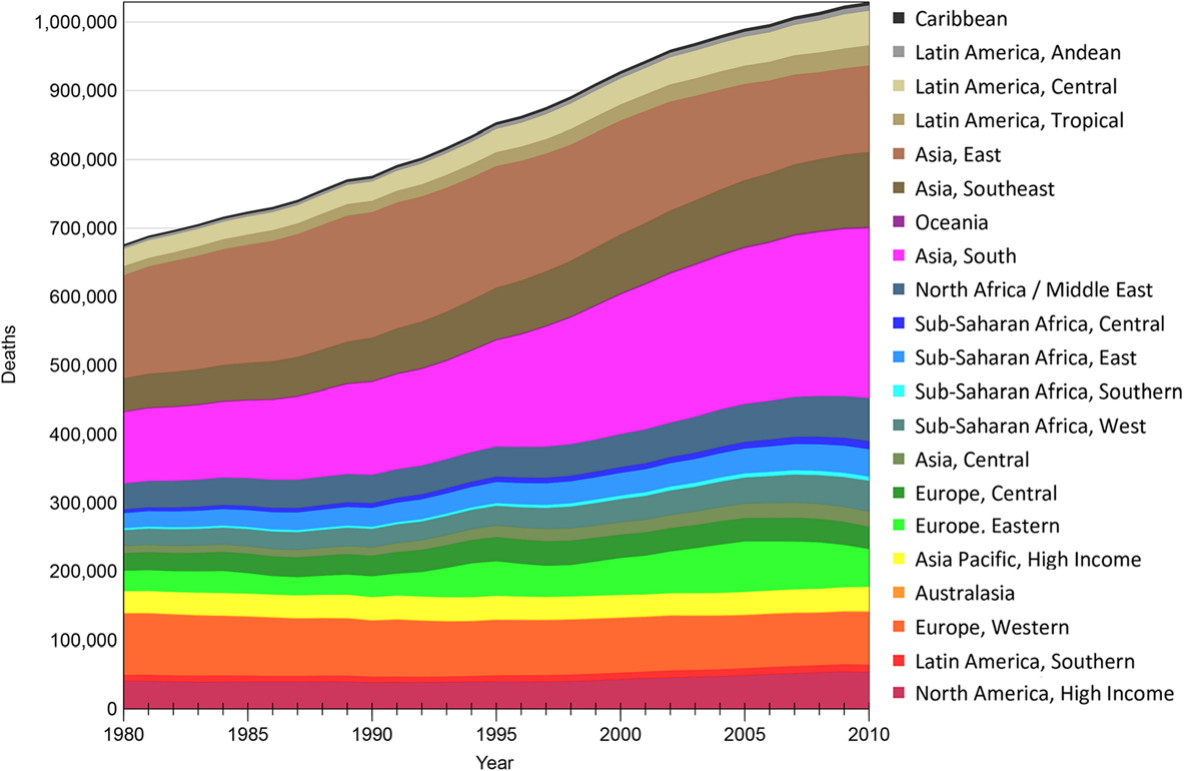
**Liver cirrhosis deaths from 1980 to 2010 by region.**

Global liver cirrhosis deaths increased monotonically from just over 676,000 (676,079:95% uncertainty interval: 452,863 to 1,004,530) deaths in 1980, or 1.54% of global deaths, to more than one million (1,029,042: 670,216 to 1,554,530) deaths in 2010, or 1.95% of the global total (Table 3, Figure 3). There were 752,100 (643,599 to 880,282) liver cancer deaths and 307,661 (268,166 to 356,476) deaths due to acute hepatitis in 2010 [2]. By comparison, there were an estimated 1,465,807 (1,334,541 to 1,606,464) HIV/AIDS deaths, 1,527,260 (1,126,448 to 1,779,624) deaths from lung cancer, 714,492 (627,825 to 822,475) colorectal cancer deaths, 7,025,343 (6,573,310 to 7,427,108) deaths due to ischemic heart disease and 5,870,446 (5,301,481 to 6,275,826) from cerebrovascular disease [2]. On average, there were twice as many liver cirrhosis male deaths as female deaths. In terms of age-standardized mortality rates^a^, liver cirrhosis decreased globally from 20.0 (95% uncertainty interval; 13.5 to 29.4) deaths per 100,000 person-years in 1980 to 15.8 (10.2 to 23.6) deaths per 100,000 person-years in 2010, a 21.6% reduction (Table 4). This was largely driven by countries in East Asia^b^ (21.2 to 8.2 deaths per 100,000; a 61.3% reduction), North Africa/Middle East (28.7 to 20.2 deaths per 100,000; a 29.6% reduction) and high-income Asia Pacific (21.2 to 10.3 deaths per 100,000; a 51.5% reduction). This trend was offset by an increase in the age-standardized liver cirrhosis mortality rate in South Asia (18.8 to 21.3 deaths per 100,000; a 12.8% increase), Central Asia (26.3 to 33.7 deaths per 100,000; a 28.4% increase) and Eastern Europe (12.6 to 20.0 deaths per 100,000; a 58.5% increase) (Table 4).

**Table 3:** **Deaths (95% uncertainty intervals) for 1980, 1990, 2000, 2010**

**Region/Country**	**1980**	**1990**	**2000**	**2010**
**Asia Pacific, High Income**				
Brunei Darussalam	9 (6–15)	10 (6–15)	10 (7–16)	15 (9–22)
Japan	19,029 (14,313-23,447)	20,697 (15,985-24,666)	21,255 (17,586-28,013)	25,693 (20,372-32,833)
Korea, Republic of	12,613 (9,091-16,460)	12,920 (9,716-15,440)	11,450 (9,255-14,694)	9,761 (7,398-14,376)
Singapore	168 (115–216)	160 (118–200)	145 (114–192)	170 (125–236)
**Asia, Central**				
Armenia	310 (232–413)	407 (325–555)	513 (399–646)	752 (468–996)
Azerbaijan	1,115 (859–1,507)	1,296 (1,056-1,697)	1,765 (1,326-2,104)	2,254 (1,672-2,941)
Georgia	1,072 (787–1,377)	1,316 (1,028-1,659)	1,052 (824–1,347)	1,176 (849–1,651)
Kazakhstan	2,664 (1,998-3,426)	2,744 (2,187-3,757)	4,288 (3,436-5,389)	4,989 (3,225-6,585)
Kyrgyzstan	813 (609–1,141)	953 (767–1,302)	1,527 (1,151-1,846)	1,780 (1,184-2,319)
Mongolia	463 (274–742)	514 (320–786)	758 (469–1,175)	1,004 (607–1,599)
Tajikistan	575 (429–769)	738 (592–965)	954 (716–1,189)	1,263 (860–1,760)
Turkmenistan	646 (484–844)	778 (614–979)	1,344 (974–1,783)	1,383 (888–2,098)
Uzbekistan	3,162 (2,395-4,184)	3,795 (3,085-4,894)	6,312 (4,767-7,815)	7,821 (5,243-11,482)
**Asia, East**				
China	144,316 (85,432-232,946)	174,878 (115,884-222,366)	155,645 (119,644-196,756)	114,352 (77,031-197,745)
Korea, Democratic People’s Republic	2,278 (1,293-3,782)	3,225 (1,900-5,190)	4,143 (2,421-6,707)	4,934 (2,851-7,929)
Taiwan	3,723 (2,267-5,862)	4,984 (3,075-7,679)	5,598 (3,532-8,456)	6,243 (3,932-9,377)
**Asia, South**				
Bangladesh	14,520 (9,789-21,278)	22,303 (15,286-31,403)	19,562 (13,646-27,524)	23,843 (15,699-34,825)
Bhutan	71 (35–132)	85 (44–154)	76 (40–137)	102 (54–185)
India	77,741 (52,196-116,746)	95,931 (71,676-134,482)	156,383 (98,953-207,554)	188,575 (109,748-303,989)
Nepal	1,831 (1,047-3,069)	2,262 (1,368-3,641)	2,568 (1,582-4,038)	3,175 (1,954-5,024)
Pakistan	10,324 (6,129-16,651)	14,453 (8,249-24,503)	24,542 (13,529-44,344)	31,373 (16,325-61,028)
**Asia, Southeast**				
Cambodia	649 (387–1,033)	814 (484–1,293)	1,186 (715–1,885)	1,538 (936–2,393)
Indonesia	16,925 (10,531-26,463)	25,115 (15,652-37,890)	35,970 (23,507-53,062)	49,224 (33,005-71,073)
Lao People’s Democratic Republic	508 (259–935)	559 (287–1,028)	650 (329–1,224)	752 (378–1,423)
Malaysia	860 (578–1,208)	997 (639–1,499)	1,163 (848–1,586)	1,488 (977–2,186)
Maldives	8 (4–17)	9 (4–17)	7 (4–12)	8 (4–12)
Mauritius	156 (114–235)	194 (148–253)	251 (173–315)	263 (196–341)
Myanmar	9,382 (4,307-18,218)	11,814 (5,267-22,841)	14,916 (6,173-30,585)	17,411 (6,684-37,173)
Philippines	2,623 (1,995-3,691)	3,316 (2,636-4,660)	6,509 (4,603-7,924)	9,173 (6,458-11,939)
Seychelles	4 (3–7)	6 (4–10)	9 (6–13)	14 (9–20)
Sri Lanka	1,047 (785–1,387)	1,123 (834–1,758)	2,942 (1,516-4,190)	3,435 (1,648-5,191)
Thailand	6,959 (4,907-9,214)	9,176 (6,312-11,770)	9,240 (6,923-13,738)	11,507 (7,738-16,920)
Timor-Leste	39 (22–64)	46 (27–76)	48 (28–78)	61 (36–97)
Viet Nam	8,164 (4,548-13,536)	9,878 (5,732-16,101)	12,892 (7,745-20,638)	14,098 (8,357-23,161)
**Australasia**				
Australia	1,397 (1,081-1,823)	1,336 (1,078-1,716)	1,417 (1,098-1,750)	1,628 (1,197-2,091)
New Zealand	172 (128–225)	174 (137–219)	191 (144–235)	208 (150–270)
**Caribbean**				
Antigua and Barbuda	7 (5–10)	6 (4–7)	6 (5–8)	8 (6–11)
Bahamas	37 (24–52)	31 (22–42)	24 (16–35)	21 (13–33)
Barbados	22 (16–31)	27 (20–35)	33 (23–44)	32 (22–43)
Belize	9 (7–13)	10 (7–14)	25 (17–34)	32 (20–44)
Cuba	734 (577–969)	1,023 (809–1,276)	1,217 (957–1,519)	1,393 (1,027-1,803)
Dominica	5 (3–7)	6 (4–8)	7 (5–9)	7 (5–10)
Dominican Republic	738 (565–928)	1,134 (837–1,363)	1,250 (1,007-1,603)	1,576 (1,189-2,302)
Grenada	8 (5–13)	8 (6–12)	12 (8–16)	12 (9–17)
Guyana	141 (98–201)	146 (108–195)	153 (108–202)	171 (117–235)
Haiti	655 (411–1,000)	782 (482–1,220)	823 (557–1,183)	972 (610–1,473)
Jamaica	112 (82–145)	94 (70–125)	118 (79–174)	130 (86–191)
Saint Lucia	18 (13–25)	20 (14–26)	19 (14–26)	18 (12–26)
Saint Vincent and the Grenadines	7 (5–10)	8 (6–12)	12 (8–16)	11 (8–14)
Suriname	47 (34–63)	45 (33–58)	46 (34–61)	40 (28–54)
Trinidad and Tobago	139 (87–185)	120 (91–153)	139 (107–191)	144 (105–199)
**Europe, Central**				
Albania	127 (75–206)	145 (98–203)	148 (91–226)	192 (114–306)
Bosnia and Herzegovina	580 (383–848)	606 (457–792)	603 (398–882)	654 (417–988)
Bulgaria	1,467 (1,163-2,036)	1,808 (1,428-2,267)	1,727 (1,356-2,183)	1,678 (1,225-2,115)
Croatia	1,488 (1,086-1,995)	1,555 (1,250-1,914)	1,506 (1,216-1,851)	1,292 (1,011-1,625)
Czech Republic	2,202 (1,631-2,995)	2,136 (1,739-2,868)	2,083 (1,631-2,543)	2,139 (1,539-2,641)
Hungary	4,031 (3,102-6,023)	5,773 (4,755-7,167)	6,306 (4,693-7,379)	4,800 (3,749-5,834)
Macedonia	111 (77–153)	126 (93–167)	160 (121–205)	151 (109–206)
Montenegro	22 (12–35)	24 (14–38)	30 (19–46)	27 (17–42)
Poland	5,136 (3,939-6,717)	5,781 (4,719-7,920)	6,981 (5,260-8,881)	7,604 (5,223-9,874)
Romania	7,083 (5,737-9,379)	8,450 (7,000-10,818)	10,904 (8,543-12,967)	10,558 (8,090-12,547)
Serbia	822 (537–1,212)	854 (607–1,185)	1,041 (833–1,378)	951 (721–1,203)
Slovakia	1,387 (997–1,835)	1,574 (1,211-2,028)	1,444 (1,162-1,790)	1,524 (1,164-1,870)
Slovenia	856 (641–1,108)	767 (616–945)	710 (574–875)	583 (449–729)
**Europe, Eastern**				
Belarus	807 (609–1,058)	852 (666–1,211)	1,603 (1,218-2,021)	2,341 (1,268-3,134)
Estonia	135 (97–206)	134 (97–212)	268 (183–332)	205 (108–277)
Latvia	247 (187–338)	244 (188–350)	375 (276–460)	350 (207–462)
Lithuania	354 (259–518)	378 (283–595)	627 (465–764)	894 (466–1,195)
Moldova	2,704 (2,141-3,331)	3,048 (2,490-3,597)	2,880 (2,344-3,443)	3,331 (2,698-4,063)
Russian Federation	17,308 (13,570-23,156)	17,145 (13,447-24,683)	33,678 (26,450-42,092)	34,770 (20,997-45,456)
Ukraine	8,331 (6,432-11,298)	8,659 (6,837-12,325)	14,521 (11,158-17,879)	13,287 (7,915-17,374)
**Europe, Western**				
Andorra	3 (2–5)	5 (3–8)	6 (4–10)	9 (5–14)
Austria	2,572 (2,082-3,268)	2,266 (1,833-2,763)	2,050 (1,657-2,504)	1,890 (1,514-2,363)
Belgium	1,890 (1,524-2,448)	1,735 (1,400-2,128)	1,706 (1,315-2,165)	1,761 (1,310-2,285)
Cyprus	45 (27–72)	44 (27–68)	60 (40–87)	65 (45–91)
Denmark	674 (516–1,009)	789 (642–1,030)	972 (702–1,158)	920 (626–1,192)
Finland	429 (288–771)	656 (514–890)	756 (549–911)	987 (542–1,293)
France	16,684 (12,009-20,184)	12,311 (10,026-15,212)	11,997 (9,886-15,437)	11,576 (9,080-15,245)
Germany	21,740 (17,542-28,243)	20,934 (17,294-26,172)	21,361 (16,753-25,370)	19,020 (14,991-23,196)
Greece	1,401 (1,024-1,698)	1,488 (1,126-1,764)	1,312 (1,080-1,815)	1,376 (1,073-1,920)
Iceland	7 (5–9)	8 (6–10)	7 (5–9)	8 (6–11)
Ireland	212 (163–299)	197 (156–270)	273 (198–336)	346 (200–459)
Israel	358 (277–476)	417 (340–523)	639 (494–768)	606 (464–760)
Italy	20,244 (14,594-25,318)	16,935 (13,399-19,986)	13,911 (11,550-18,249)	13,539 (10,719-17,927)
Luxembourg	99 (75–130)	91 (69–116)	93 (69–115)	88 (63–113)
Malta	37 (27–49)	30 (22–38)	35 (26–45)	37 (27–48)
Netherlands	1,029 (752–1,518)	1,187 (884–1,695)	1,512 (1,080-2,003)	1,535 (1,046-2,062)
Norway	280 (214–397)	327 (257–418)	350 (263–423)	343 (245–434)
Portugal	3,171 (2,414-3,828)	3,027 (2,331-3,609)	2,500 (2,086-3,352)	2,409 (1,918-3,230)
Spain	9,964 (7,344-12,243)	10,091 (7,806-12,011)	9,181 (7,581-11,904)	8,853 (7,063-11,774)
Sweden	909 (691–1,242)	844 (687–1,100)	887 (688–1,086)	943 (663–1,162)
Switzerland	884 (686–1,136)	767 (625–1,024)	828 (645–1,011)	790 (587–1,012)
United Kingdom	5,426 (4,054-8,663)	5,985 (4,829-8,262)	7,944 (5,684-9,478)	8,567 (5,396-10,952)
**Latin America, Andean**				
Bolivia	873 (549–1,333)	1,064 (667–1,605)	1,364 (845–2,098)	1,495 (901–2,360)
Ecuador	793 (603–1,152)	1,145 (918–1,468)	1,661 (1,255-2,005)	2,062 (1,471-2,579)
Peru	1,774 (1,408-2,383)	2,156 (1,756-3,004)	3,472 (2,546-4,107)	4,381 (3,213-5,601)
**Latin America, Central**				
Colombia	1,503 (1,159-2,040)	2,003 (1,646-2,653)	2,880 (2,217-3,510)	3,674 (2,672-4,600)
Costa Rica	196 (146–280)	308 (247–394)	503 (382–607)	687 (493–858)
El Salvador	567 (404–865)	596 (448–863)	939 (652–1,189)	1,087 (773–1,464)
Guatemala	1,035 (674–1,821)	1,605 (1,266-2,064)	2,283 (1,701-2,796)	3,201 (2,268-4,154)
Honduras	302 (213–417)	451 (323–611)	665 (404–1,051)	893 (539–1,433)
Mexico	20,024 (16,207-25,646)	21,035 (17,136-26,026)	27,583 (21,630-32,585)	36,157 (28,929-45,043)
Nicaragua	349 (242–509)	378 (291–532)	668 (489–817)	938 (667–1,231)
Panama	135 (102–178)	180 (135–236)	122 (90–160)	286 (206–372)
Venezuela	1,278 (995–1,688)	1,759 (1,366-2,168)	2,308 (1,808-2,911)	3,527 (2,581-4,622)
**Latin America, Southern**				
Argentina	5,089 (3,918-6,362)	4,693 (3,863-6,133)	5,720 (4,572-7,075)	6,520 (4,879-8,038)
Chile	3,669 (2,837-4,565)	3,554 (2,948-4,501)	3,700 (2,926-4,469)	4,082 (3,133-5,176)
Uruguay	414 (325–554)	471 (371–588)	496 (383–614)	489 (359–645)
**Latin America, Tropical**				
Brazil	13,394 (10,329-18,395)	16,615 (13,149-21,424)	23,325 (17,877-28,906)	29,075 (21,227-36,547)
Paraguay	170 (119–235)	205 (149–276)	350 (251–458)	556 (372–798)
**North Africa/Middle East**				
Afghanistan	1,863 (998–3,238)	1,581 (852–2,762)	3,085 (1,611-5,517)	3,688 (1,952-6,493)
Algeria	1,432 (782–2,491)	788 (441–1,325)	1,031 (633–1,611)	1,248 (800–1,873)
Bahrain	14 (9–21)	22 (14–32)	28 (19–37)	34 (24–48)
Egypt	23,895 (14,900-39,647)	27,385 (18,873-39,451)	30,514 (24,370-37,617)	41,844 (32,811-50,462)
Iran, Islamic Republic of	1,985 (1,313-2,780)	2,333 (1,520-3,421)	2,540 (1,916-3,551)	2,801 (1,785-4,295)
Iraq	505 (283–845)	608 (342–1,018)	756 (435–1,230)	968 (549–1,603)
Jordan	244 (139–413)	194 (114–313)	249 (159–376)	321 (211–471)
Kuwait	55 (39–77)	44 (32–64)	75 (55–98)	128 (85–171)
Lebanon	153 (92–237)	152 (92–237)	206 (126–322)	235 (144–365)
Libyan Arab Jamahiriya	135 (75–224)	177 (96–296)	253 (148–404)	385 (226–616)
Morocco	2,550 (1,496-4,254)	2,503 (1,524-4,046)	2,819 (1,813-4,226)	3,363 (2,285-4,824)
Occupied Palestinian Territory	133 (67–250)	150 (80–268)	180 (106–300)	254 (159–395)
Oman	71 (37–125)	78 (45–129)	73 (46–112)	172 (113–255)
Qatar	11 (5–19)	13 (7–23)	21 (13–33)	37 (23–58)
Saudi Arabia	533 (307–890)	744 (447–1,204)	987 (614–1,528)	1,023 (637–1,601)
Syrian Arab Republic	437 (290–633)	626 (378–1,014)	556 (340–889)	559 (362–861)
Tunisia	340 (203–531)	385 (232–595)	539 (332–838)	674 (401–1,079)
Turkey	2,716 (1,774-3,767)	2,680 (1,982-3,402)	2,794 (2,174-3,674)	3,130 (2,313-4,350)
United Arab Emirates	53 (24–101)	99 (53–171)	148 (84–250)	269 (140–479)
Yemen	748 (290–1,694)	841 (322–1,969)	1,306 (536–3,064)	1,692 (694–3,948)
**North America, High Income**				
Canada	3,155 (2,387-3,907)	2,957 (2,422-3,741)	3,376 (2,721-4,144)	3,830 (2,901-4,925)
United States	37,419 (29,264-46,791)	35,496 (29,091-44,976)	39,663 (31,542-48,977)	49,538 (36,338-61,188)
**Oceania**				
Fiji	50 (26–99)	61 (34–104)	74 (45–116)	72 (43–116)
Kiribati	14 (7–27)	17 (10–29)	18 (10–31)	19 (10–36)
Marshall Islands	4 (3–8)	6 (3–9)	6 (4–11)	8 (4–13)
Micronesia, Federated States of	14 (7–29)	19 (9–37)	19 (9–36)	19 (9–37)
Papua New Guinea	620 (313–1,175)	971 (479–1,841)	1,379 (636–2,665)	1,820 (833–3,576)
Samoa	17 (10–28)	23 (14–38)	22 (13–37)	21 (12–35)
Solomon Islands	41 (19–78)	57 (27–109)	67 (32–128)	89 (41–175)
Tonga	13 (7–21)	13 (8–20)	14 (9–21)	15 (9–23)
Vanuatu	19 (7–39)	28 (12–57)	31 (15–61)	37 (18–74)
**Sub-Saharan Africa, Central**				
Angola	1,003 (489–1,895)	1,348 (639–2,700)	1,511 (711–2,967)	1,949 (946–3,660)
Central African Republic	398 (211–683)	575 (314–958)	729 (396–1,229)	997 (516–1,733)
Congo	274 (154–446)	404 (236–646)	543 (315–880)	677 (392–1,090)
Congo, the Democratic Republic of	3,185 (1,812-5,312)	4,011 (2,275-6,721)	4,989 (2,774-8,399)	7,476 (4,102-12,774)
Equatorial Guinea	30 (8–65)	55 (15–128)	48 (13–99)	75 (18–159)
Gabon	165 (83–292)	195 (104–333)	252 (138–426)	375 (199–638)
**Sub-Saharan Africa, East**				
Burundi	836 (231–2,017)	1,072 (311–2,484)	1,108 (329–2,588)	1,355 (466–2,975)
Comoros	29 (6–61)	34 (7–72)	40 (8–83)	59 (12–123)
Djibouti	26 (13–47)	50 (25–92)	77 (38–141)	105 (50–198)
Eritrea	339 (156–599)	331 (152–594)	332 (150–593)	518 (232–946)
Ethiopia	8,048 (4,850-13,046)	9,881 (6,095-15,465)	10,683 (6,632-16,491)	12,585 (7,712-19,507)
Kenya	1,127 (586–1,943)	1,391 (727–2,401)	1,996 (1,009-3,631)	2,715 (1,400-4,762)
Madagascar	1,224 (665–2,101)	1,393 (763–2,379)	1,581 (866–2,679)	2,129 (1,144-3,673)
Malawi	878 (455–1,557)	1,309 (689–2,293)	2,179 (1,157-3,738)	2,671 (1,459-4,560)
Mozambique	1,457 (656–3,029)	1,600 (730–3,327)	2,094 (1,072-4,054)	2,625 (1,316-5,252)
Rwanda	977 (441–1,823)	1,231 (607–2,167)	1,077 (537–1,877)	888 (462–1,506)
Somalia	633 (292–1,232)	692 (316–1,359)	795 (368–1,571)	1,002 (452–1,979)
Sudan	1,556 (703–2,928)	1,924 (927–3,426)	2,202 (1,106-3,849)	2,738 (1,368-4,750)
Tanzania, United Republic of	2,063 (1,184-3,500)	2,769 (1,666-4,416)	3,024 (1,981-4,422)	3,690 (2,236-5,805)
Uganda	1,912 (867–3,624)	2,545 (1,149-4,934)	3,921 (1,839-7,315)	4,891 (2,356-8,929)
Zambia	738 (405–1,262)	1,191 (663–2,017)	1,937 (1,069-3,292)	1,973 (1,127-3,276)
**Sub-Saharan Africa, Southern**				
Botswana	37 (22–61)	47 (27–76)	108 (55–188)	94 (47–164)
Lesotho	77 (38–138)	92 (45–161)	143 (67–258)	194 (88–353)
Namibia	48 (25–83)	62 (33–104)	124 (65–208)	134 (72–222)
South Africa	2,098 (1,288-3,291)	2,430 (1,591-3,511)	3,623 (2,702-4,836)	3,266 (2,174-5,148)
Swaziland	58 (26–132)	73 (32–167)	167 (72–379)	238 (101–533)
Zimbabwe	833 (464–1,336)	693 (437–1,031)	1,217 (665–2,016)	2,302 (1,271-3,887)
**Sub-Saharan Africa, West**				
Benin	542 (287–1,015)	557 (285–1,026)	750 (404–1,344)	963 (537–1,715)
Burkina Faso	1,123 (597–2,117)	1,249 (670–2,206)	1,656 (899–2,906)	2,264 (1,232-4,062)
Cameroon	2,235 (801–6,085)	2,180 (856–5,133)	2,791 (990–6,218)	3,700 (1,288-8,267)
Cape Verde	20 (6–41)	23 (5–49)	25 (3–57)	34 (4–78)
Chad	622 (340–1,122)	704 (394–1,210)	1,012 (547–1,825)	1,394 (729–2,560)
Côte d’Ivoire	1,219 (656–2,185)	1,856 (1,006-3,333)	3,448 (1,833-6,154)	4,258 (2,307-7,488)
Gambia	86 (39–178)	100 (45–206)	127 (53–271)	156 (63–326)
Ghana	1,243 (557–2,383)	1,757 (928–2,994)	2,210 (1,327-3,512)	4,060 (2,364-6,478)
Guinea	631 (332–1,178)	700 (375–1,242)	946 (510–1,702)	1,157 (633–2,095)
Guinea-Bissau	137 (64–267)	148 (69–285)	166 (71–342)	216 (92–450)
Liberia	210 (109–399)	240 (129–421)	315 (172–547)	470 (262–824)
Mali	1,605 (797–3,169)	1,406 (724–2,638)	1,455 (763–2,595)	1,703 (912–2,987)
Mauritania	179 (97–326)	212 (119–367)	264 (146–470)	373 (205–670)
Niger	858 (437–1,651)	917 (462–1,722)	1,132 (572–2,145)	1,457 (771–2,685)
Nigeria	10,411 (4,951-19,412)	12,719 (6,263-24,695)	15,185 (6,851-30,731)	19,854 (8,489-40,832)
Sao Tome and Principe	12 (6–22)	13 (6–23)	14 (6–26)	14 (6–28)
Senegal	652 (314–1,209)	705 (334–1,308)	851 (349–1,641)	1,080 (445–2,122)
Sierra Leone	742 (373–1,470)	774 (416–1,414)	697 (373–1,229)	945 (515–1,634)
Togo	418 (226–767)	475 (265–843)	563 (310–996)	795 (432–1,430)
**Global**	676,079 (452,863-1,004,530)	776,054 (547,470-1,074,719)	928,117 (654,382-1,270,701)	1,029,042 (670,216-1,554,530)

**Table 4:** **Global, region, and country-level age standardized mortality rate (per 100,000) for both sexes and percent change (Δ)**

**Country**	**1980**	**1990**	**2000**	**2010**	**% Δ 1980 to 2010**	**% Δ 1990 to 2010**
**Asia Pacific, High Income**	21.2	16.8	12.6	10.3	−51.5	−38.9
Brunei Darussalam	10.3	7.8	6.5	5.8	−44.2	−25.8
Japan	15.3	12.1	9.4	8.6	−43.4	−28.4
Korea, Republic of	52.3	39	25.1	15.2	−70.9	−61
Singapore	11.2	7.7	5.1	4.4	−60.9	−43.2
**Asia, Central**	26.3	25.4	33.7	33.7	28.4	32.7
Armenia	13.5	13.7	15.5	18.8	40.1	37.1
Azerbaijan	25.4	28.4	32.3	27.3	7.5	−3.9
Georgia	20.1	21.1	17.4	18.9	−6	−10.2
Kazakhstan	23.5	20.5	31.9	32.5	38.3	58.4
Kyrgyzstan	31	30.9	44.2	46.1	48.7	49.1
Mongolia	44	40.4	56	55.1	25.3	36.4
Tajikistan	23.9	24	29.3	31.7	32.4	31.8
Turkmenistan	37.7	36.2	47.6	39.3	4.5	8.6
Uzbekistan	31.2	30.9	40.6	40	28.2	29.4
**Asia, East**	21.2	19.9	14	8.2	−61.3	−58.8
China	21	19.7	13.6	7.7	−63.1	−60.6
Korea, Democratic People’s Republic	21.2	20	19.2	18.7	−11.9	−6.5
Taiwan	34.4	31.6	25.4	20.2	−41.3	−36.3
**Asia, South**	18.8	19.4	22.9	21.3	12.8	9.7
Bangladesh	31.3	38.8	26.1	24.2	−22.8	−37.7
Bhutan	27.2	24.8	21.9	20.8	−23.8	−16.4
India	17.1	16.9	21.9	20.2	17.9	19.4
Nepal	19.9	20.2	18.3	17.1	−14.4	−15.4
Pakistan	21.7	22.4	29.3	28.5	31.3	27.5
**Asia, Southeast**	22.1	22.5	22.7	21.3	−3.3	−5
Cambodia	18.3	16.7	16.6	16.3	−11.4	−2.5
Indonesia	19.8	22.4	24.3	24.8	25.1	10.6
Lao People’s Democratic Republic	26.3	23.8	22.1	19.6	−25.5	−17.7
Malaysia	11.5	10	8.2	7.2	−37.6	−28.8
Maldives	8.3	6.7	4.3	3.7	−55	−44.5
Mauritius	27.1	26.2	24.4	19.2	−29.2	−26.8
Myanmar	45.6	47.7	47.6	42.6	−6.7	−10.7
Philippines	10.9	10.1	15.1	15.6	43	53.7
Seychelles	13.4	16.8	23.9	31.2	133.1	85.8
Sri Lanka	11	8.8	17.6	15.9	44.7	80.4
Thailand	24.1	22.4	15.6	14.6	−39.5	−34.8
Timor-Leste	14.5	13.4	12	11	−24.3	−18.1
Viet Nam	24.3	24	23.6	18.5	−23.7	−22.8
**Australasia**	8.4	6.4	5.2	4.6	−45.5	−28.1
Australia	9.1	6.8	5.5	4.9	−46.1	−28
New Zealand	5.2	4.3	3.7	3.1	−41.3	−28.9
**Caribbean**	14.5	15.2	13.7	12.7	−12.6	−16.3
Antigua and Barbuda	14.4	11.6	9.9	9.8	−32	−15.3
Bahamas	30.1	18.4	9	6.1	−79.9	−67
Barbados	9.4	10.8	10.3	8.5	−9.6	−21.9
Belize	11.5	10.1	19.5	17.2	49.9	70.5
Cuba	8.7	9.8	9.7	8.8	1.2	−10
Dominica	9.4	10.6	11.4	10.4	11.6	−1.5
Dominican Republic	26.9	28.9	21.6	19.6	−26.9	−32
Grenada	12.9	11	14.8	14	8.6	27.6
Guyana	33.5	32.2	29.9	30.7	−8.4	−4.9
Haiti	19.5	19.3	17	16.4	−16	−15.4
Jamaica	7	4.8	5.1	4.9	−30.2	2.7
Saint Lucia	25.8	19.7	15.5	11.4	−56	−42.3
Saint Vincent and the Grenadines	10.7	11.6	13.8	10.8	1.8	−6.8
Suriname	21.8	16.4	13	8.9	−59.4	−46
Trinidad and Tobago	18.9	14.2	13.3	11	−41.9	−22.4
**Europe, Central**	19.3	20.6	21.4	18.4	−4.9	−10.8
Albania	7.4	6.5	5.4	5.4	−26.1	−16.8
Bosnia and Herzegovina	18.6	14.9	13	11.4	−38.9	−23.7
Bulgaria	12.9	14.8	14.1	13.6	5.3	−8
Croatia	28	26.4	22.4	17.8	−36.5	−32.8
Czech Republic	17.3	16.5	14.8	13.2	−23.4	−19.8
Hungary	29.9	43.7	46	32.8	9.5	−25.1
Macedonia	7.3	6.8	6.9	5.5	−24.8	−19.6
Montenegro	4	3.7	4.1	3.2	−20.3	−14.1
Poland	13.2	13.3	14.5	13.8	4	3.6
Romania	28.6	30.5	37.1	33.5	17	9.8
Serbia	8.3	7.4	7.6	6.7	−19.5	−10.3
Slovakia	26.7	28.1	22.9	20.6	−22.7	−26.4
Slovenia	41	31.8	25.8	17.7	−56.7	−44.2
**Europe, Eastern**	12.6	11.3	19.8	20	58.5	77.5
Belarus	7.5	6.9	12.9	18.2	143.3	165.8
Estonia	7.7	6.8	14.8	10.8	40.5	57.8
Latvia	7.9	7.1	11.6	10.9	37.3	53.8
Lithuania	9.3	8.8	14.6	20.2	117.7	130
Moldova	70	68.2	62	71.2	1.7	4.3
Russian Federation	11.3	9.7	18.5	18.6	64.4	91.4
Ukraine	14	13	23.2	22.1	58.3	70
**Europe, Western**	17.9	14.7	12.6	10.2	−42.9	−30.5
Andorra	6.9	6.7	5.8	5.2	−24.4	−22.8
Austria	25.5	21.6	17.4	13.4	−47.5	−38
Belgium	13.8	11.6	10.8	9.3	−32.6	−20
Cyprus	6.7	5.6	6.4	6	−11.6	6.6
Denmark	10.6	11.8	12.8	10.6	0.2	−10.2
Finland	7.7	10.2	10.2	11.8	53.3	15.5
France	24.5	16.3	13.8	11.2	−54.3	−31.2
Germany	19.8	18.2	16.4	12.4	−37.6	−32.1
Greece	10.4	9.3	6.9	6	−41.8	−34.7
Iceland	2.7	2.5	2	1.7	−35	−30.4
Ireland	5.7	4.8	5.9	6	5	25.5
Israel	10.3	8.5	8.8	6.3	−38.6	−26
Italy	26.8	19.1	13	10.2	−61.9	−46.6
Luxembourg	20.7	17.6	15.4	11.4	−44.8	−35.2
Malta	11.3	7.3	6.7	5.3	−52.6	−26.7
Netherlands	6.1	5.9	6.4	5.2	−14	−10.9
Norway	5.2	5.4	5.1	4.2	−19.3	−22.4
Portugal	27.6	23.1	16.4	13.6	−50.8	−41.1
Spain	22.3	18.8	14	10.8	−51.5	−42.4
Sweden	7.7	6.3	5.7	5.4	−30.6	−14.6
Switzerland	10.5	8.2	7.5	5.8	−44.9	−29.4
United Kingdom	6.9	7.1	9.2	9	31.2	27.7
**Latin America, Andean**	20.5	19.5	21.6	19.6	−4.1	0.5
Bolivia	29.4	28.1	27.9	23.5	−20.1	−16.5
Ecuador	17.6	19.1	19.9	17.8	1	−6.9
Peru	19.1	17.2	20.5	19.4	1.4	12.9
**Latin America, Central**	35.2	29.4	28.6	27.5	−21.9	−6.5
Colombia	10.3	10.6	11.4	10.4	1.6	−1.2
Costa Rica	13.7	15.8	17.8	16.6	21.3	4.5
El Salvador	21.4	18.1	22.4	21.9	2.3	20.7
Guatemala	26.6	32.7	36	37.4	40.7	14.4
Honduras	18.6	20.3	21.2	20.4	9.3	0.3
Mexico	53.4	42.2	40.3	38.3	−28.3	−9.1
Nicaragua	24	20	25.6	26	8	29.6
Panama	11.6	11.8	5.4	9.6	−17.6	−18.9
Venezuela	17.2	16.5	14.6	15.3	−10.7	−7
**Latin America, Southern**	24	18.9	17.4	15.9	−33.8	−16
Argentina	18.9	14.5	14.7	14	−26.1	−3.7
Chile	45.9	34.9	26.9	21.8	−52.5	−37.5
Uruguay	12.3	12.3	11.5	10.2	−17.3	−16.9
**Latin America, Tropical**	17.3	16.3	16.9	15.8	−8.8	−3.3
Brazil	17.5	16.5	17.1	15.9	−9.2	−3.9
Paraguay	10	8.9	10.9	12.5	24.7	39.6
**North Africa/Middle East**	28.7	25	21.5	20.2	−29.6	−19.1
Afghanistan	26.2	25.6	29.7	26.9	2.9	5.5
Algeria	11.4	5.3	5.7	5.1	−55.1	−4.2
Bahrain	9.4	10.3	8.5	5.2	−45.1	−49.7
Egypt	98	87.3	72.9	72.7	−25.9	−16.8
Iran, Islamic Republic of	8.8	7.8	6.6	5.2	−40.7	−33.5
Iraq	6.2	6.4	6	6	−3.2	−5.9
Jordan	20.9	11.3	10.5	10	−52	−11.3
Kuwait	10.3	5.5	7.9	9.7	−5.7	75.3
Lebanon	8.5	7.6	6.9	6.2	−27.5	−19.5
Libyan Arab Jamahiriya	10.4	9.4	9.5	9.9	−5.4	5
Morocco	21.4	17.8	15.8	14.3	−33.4	−19.8
Occupied Palestinian Territory	17.3	15.4	12.3	13.1	−24.7	−15
Oman	11.5	10.1	7.9	13.1	14.7	29.8
Qatar	14.3	8.5	10	7.4	−47.8	−12.8
Saudi Arabia	12.3	11.6	11.4	8	−34.8	−30.7
Syrian Arab Republic	9.5	10.2	6.8	4.7	−50.3	−53.7
Tunisia	9.6	7.6	7.6	7.2	−24.5	−5.2
Turkey	10.3	8	6.5	5.2	−49	−34.7
United Arab Emirates	15.6	14.7	13.8	11.1	−28.3	−24.2
Yemen	19.4	17.1	16.7	16.6	−14.4	−3.2
**North America, High Income**	14.6	11.5	10.5	10.8	−25.8	−6.3
Canada	12.3	8.9	7.9	6.9	−44.5	−23.1
United States	14.8	11.8	10.8	11.3	−23.9	−4.8
**Oceania**	38.4	43.4	44	41.5	8.1	−4.6
Fiji	15.6	15.3	14.6	11.2	−28	−26.7
Kiribati	54.8	50.6	41.8	34.7	−36.7	−31.5
Marshall Islands	35.2	28.3	24.1	21.2	−39.8	−25.3
Micronesia, Federated States of	39.1	39.9	32.1	28.5	−27	−28.5
Papua New Guinea	44.5	51.7	53.6	51	14.5	−1.4
Samoa	28.2	27	21.4	16.6	−41.1	−38.4
Solomon Islands	37.1	39	32.6	30.1	−19	−22.8
Tonga	25.2	23.1	21.7	20.3	−19.3	−12.2
Vanuatu	35.3	36.9	30.9	26.7	−24.3	−27.6
**Sub-Saharan Africa, Central**	22.9	23.1	22	24.2	5.5	4.5
Angola	24	25.4	22.1	21.6	−10.2	−15
Central African Republic	27.4	32.6	33.3	38.5	40.7	18.2
Congo	26.9	30.4	30.5	29.4	9.6	−3.2
Congo, the Democratic Republic of	21.5	20.6	19.9	22.9	6.7	11
Equatorial Guinea	18.4	24.5	15.1	16.4	−10.9	−33.1
Gabon	30.8	31.6	33.6	38.6	25.5	22.2
**Sub-Saharan Africa, East**	28	26.7	24.6	23.1	−17.5	−13.4
Burundi	37.4	36.7	35.2	32	−14.6	−13
Comoros	17.3	16.7	15.4	17	−1.8	1.8
Djibouti	17.5	19.1	21.4	21.4	21.9	12
Eritrea	24	22.1	21.3	22.8	−5.3	3
Ethiopia	44.6	41	33.3	29.6	−33.6	−27.8
Kenya	15.3	14	12.9	14.5	−4.8	3.7
Madagascar	26.1	24.1	21.6	21.1	−19.3	−12.6
Malawi	26.3	27.2	37.3	36.6	39.2	34.6
Mozambique	20.7	20.2	21.5	21	1.8	4.1
Rwanda	41.9	39.4	28.4	18.7	−55.3	−52.5
Somalia	19.3	20.7	22.1	22.2	15	7.3
Sudan	15.3	14.4	12.5	11.6	−24.2	−19.4
Tanzania, United Republic of	22.6	22.3	17.5	16.6	−26.7	−25.7
Uganda	32.3	27.3	34.3	36	11.5	31.6
Zambia	25.6	29.5	36.9	32.1	25.4	8.8
**Sub-Saharan Africa, Southern**	15.3	12.5	13.6	12.9	−15.7	3.6
Botswana	8.9	7.5	9.4	7.2	−19.7	−5
Lesotho	10.4	9.8	12.6	15.4	48.6	57.4
Namibia	8.8	8.8	12.3	10	13.7	13.9
South Africa	13.5	12.1	12.7	8.7	−35.5	−28
Swaziland	20.9	19.1	31.8	37.8	81	97.5
Zimbabwe	25.7	14.7	16.7	33.2	29.1	125.3
**Sub-Saharan Africa, West**	27.4	24.4	22.8	23.5	−14	−3.6
Benin	25.3	20.9	21.3	21	−16.8	0.5
Burkina Faso	28.9	26	26.8	26.7	−7.7	2.6
Cameroon	42.4	32.5	30.9	32.1	−24.1	−1
Cape Verde	11.4	11.1	9.7	9.8	−13.9	−12.3
Chad	22.8	20.9	22.2	23	1	10.3
Côte d’Ivoire	30.2	29	36.3	36.3	20.3	25.1
Gambia	28.5	24.4	22.1	19.8	−30.3	−18.8
Ghana	21.2	21.8	19.7	26.1	23	19.8
Guinea	24.4	21.2	19.8	21	−13.6	−0.8
Guinea-Bissau	27.3	24.8	22.6	24.2	−11.4	−2.3
Liberia	21.4	21.7	21.9	22.8	6.7	5.5
Mali	39.6	30	24.3	23.4	−40.9	−22
Mauritania	25.4	22.7	19.9	20.2	−20.4	−11.1
Niger	30.1	22.6	18.7	18.5	−38.3	−17.8
Nigeria	24.9	23.1	20.8	21	−15.7	−9.4
Sao Tome and Principe	20.3	18.7	18.2	16.9	−16.9	−9.7
Senegal	24	20.5	18.3	18.9	−21.2	−7.8
Sierra Leone	41.3	36.3	32.1	33	−20.1	−8.9
Togo	30.2	25.5	22.2	22.8	−24.3	−10.5
**Global**	20	18.6	17.8	15.7	−21.6	−15.8

**Figure 4:**
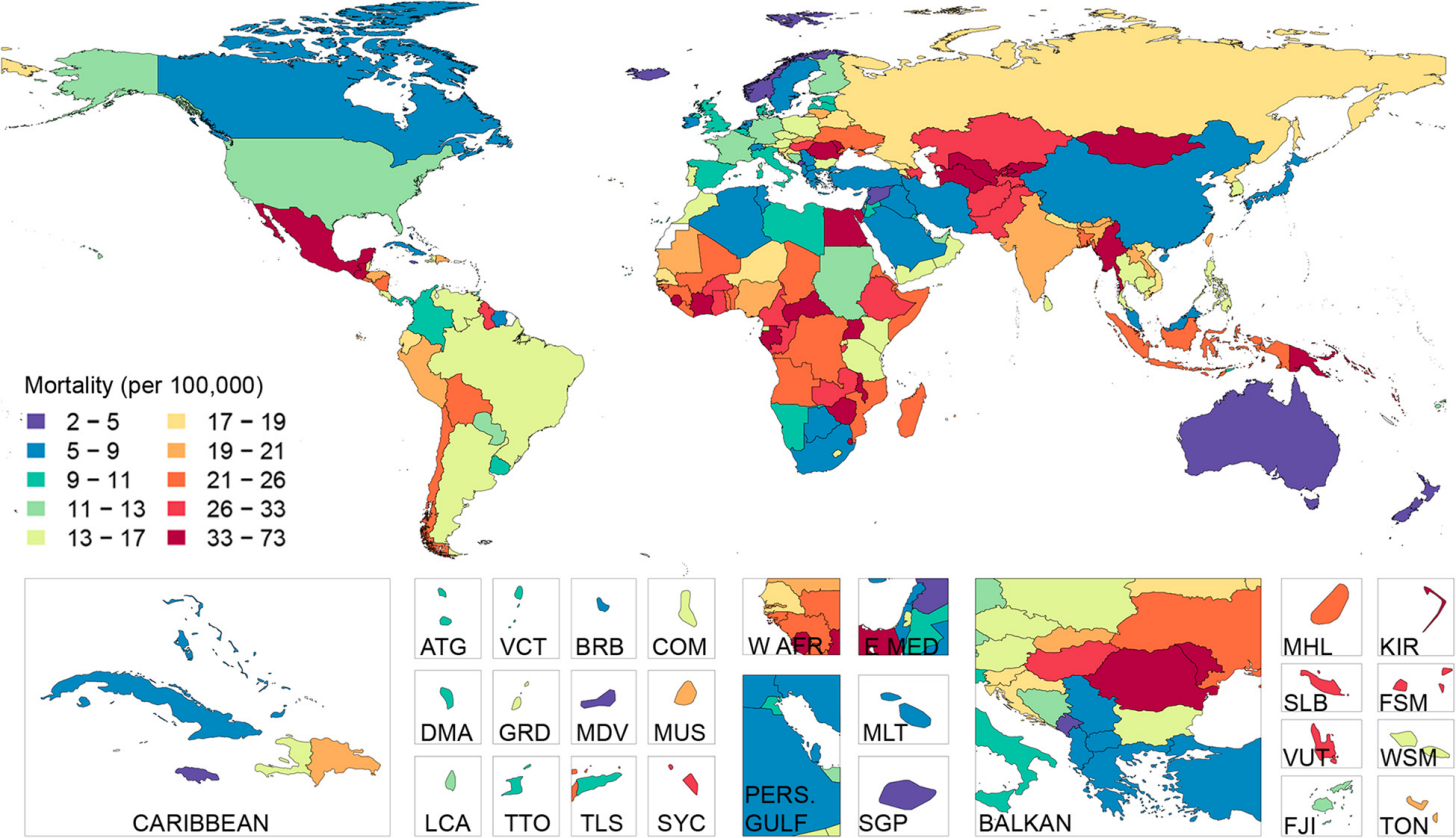
**Age-adjusted liver cirrhosis mortality (per 100,000) for both sexes in 2010.** ATG(Antigua and Barbuda), BRB(Barbados), COM(Comoros), DMA(Dominica), E Med(East Mediterranean), FJI(Fiji), FSM(Micronesia, Federated States of), GRD(Grenada), KIR(Kiribati), LCA(Saint Lucia), MDV(Maldives), MHL(Marshall Islands), MLT(Malta), MUS(Mauritius), PERS GULF(Persian Gulf), SGP(Singapore), SLB(Solomon Islands), SYC(Seychelles), TLS(Timor-Leste), TON(Tonga), TTO(Trinidad and Tobago), VCT(Saint Vincent and the Grenadines), VUT(Vanuatu), W AFR(West Africa), WSM(Samoa).

Age-standardized liver cirrhosis mortality rates and time trends in Europe followed a strong ‘East-to-West gradient’, as previously identified by Zatonski *et al*. [5] (Figure 4). In Hungary, Moldova and Romania, cirrhosis mortality rates increased to peak levels in the mid-1990s, then declined. Nonetheless, age-standardized cirrhosis mortality rates in those countries still ranked in the upper tenth percentile of the world in 2010. In Russia, the cirrhosis mortality rate increased drastically after 1990 but has declined in the last five years. Moldova is unusual compared to its neighbors due to its high cirrhosis mortality rates among females, which were similar to males (male-to-female ratio of 1.15) and caused about one in five (0.15 to 0.26) female deaths at ages 45 to 54 years for the period between 1980 and 2010. In Russia, the male-to-female cirrhosis mortality rate is also beginning to converge. This contrasts with neighboring countries where alcoholic liver cirrhosis was much more common in men; for example, in Bulgaria, Romania or Ukraine, the male-to-female mortality ratio exceeds two. Country level male to female mortality ratios and sex specific liver cirrhosis mortality for the year 2010 can be found in Additional file 7: Figure S1, Additional file 8: Figure S2 and Additional file 9: Figure S3, respectively.

Most Western European countries have succeeded in reducing mortality from cirrhosis. In Italy, France, Germany and Spain, age-standardized liver cirrhosis mortality rates in 1980 were among the upper 30th percentile globally. By 2010, cirrhosis mortality in these countries ranked in the lowest tertile globally. Since the 1970s, improvements in alcohol quality and a reduction in alcohol consumption have been the major determinants of the steady decline in cirrhosis mortality in these countries [5,36–39]. A similar picture was observed in most other countries in Western Europe, with the exception of the UK, Ireland, and Finland, where cirrhosis mortality rates have continued to increase since the late 1980s. Alcohol intake remains the most common cause of liver cirrhosis in Western Europe. A third of liver cirrhosis cases is attributable to heavy alcohol intake, this proportion being highest among all regions (Figure 2, Additional file 6: Table S6).

**Figure 5:**
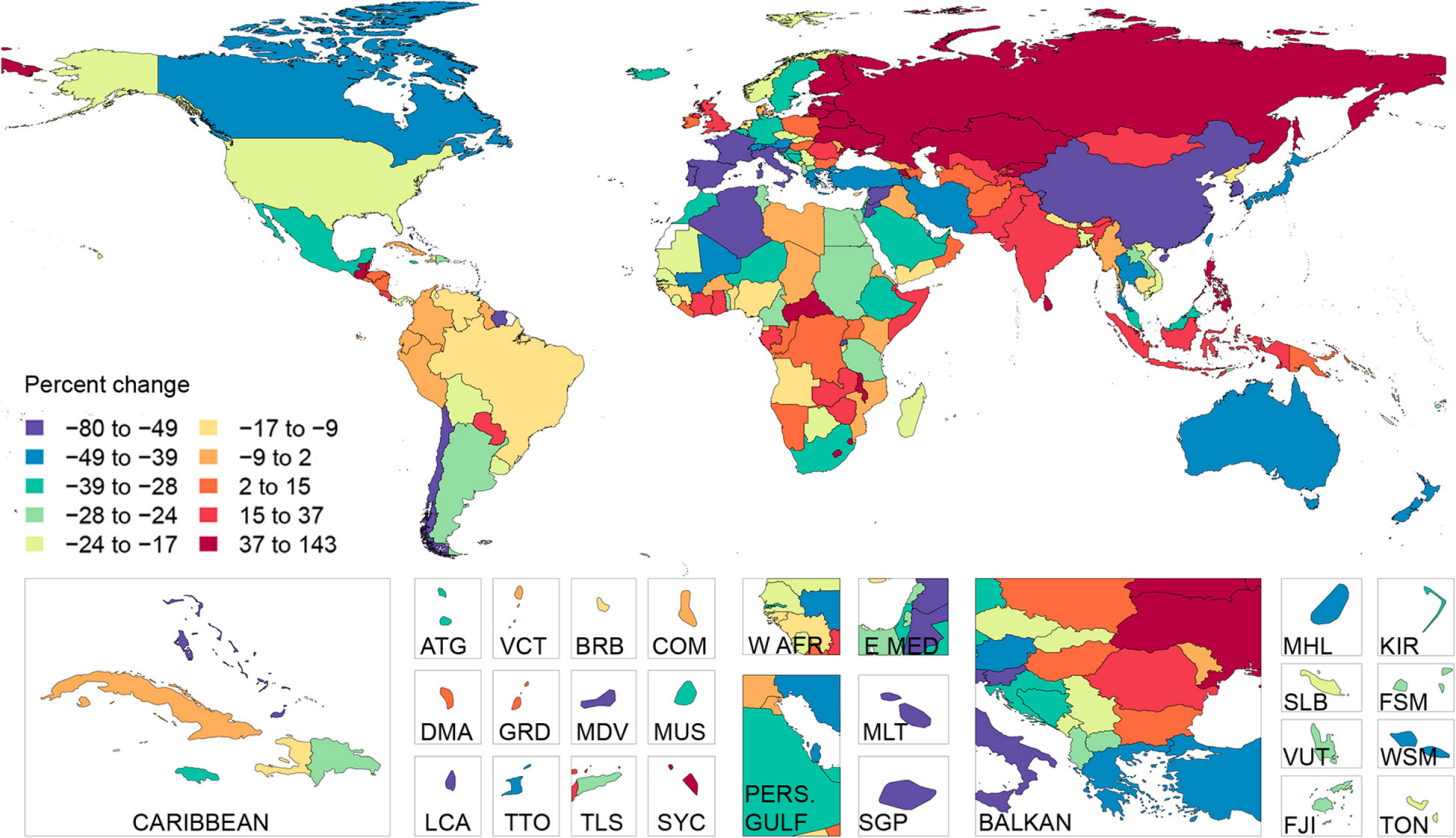
**Percent change in age-adjusted liver cirrhosis mortality between 1980 and 2010.** ATG(Antigua and Barbuda), BRB(Barbados), COM(Comoros), DMA(Dominica), E Med(East Mediterranean), FJI(Fiji), FSM(Micronesia, Federated States of), GRD(Grenada), KIR(Kiribati), LCA(Saint Lucia), MDV(Maldives), MHL(Marshall Islands), MLT(Malta), MUS(Mauritius), PERS GULF(Persian Gulf), SGP(Singapore), SLB(Solomon Islands), SYC(Seychelles), TLS(Timor-Leste), TON(Tonga), TTO(Trinidad and Tobago), VCT(Saint Vincent and the Grenadines), VUT(Vanuatu), W AFR(West Africa), WSM(Samoa).

Liver cirrhosis mortality trends vary widely among countries in Latin America (Table 4, Figure 4). Mortality rates increased in Costa Rica, Guatemala, Honduras and Paraguay, but fell in Chile, Mexico and Argentina. In 1980, age-standardized cirrhosis mortality rates in Chile and Mexico were, respectively, 53.4 (43.6 to 67.9) per 100,000 and 45.9 (35.6 to 57.0) per 100,000, the highest in the region. In 2010, Mexico remained the country with the highest cirrhosis mortality rate in the region, at 38.3 (30.7 to 47.5) per 100,000 (Table 4, Figure 5). Liver cirrhosis was the fourth-leading cause of death in Mexico in 2010, accounting for 18% of deaths in males aged 40 to 49 years.

From the early 1990s, cirrhosis deaths began to increase in many countries in Central Asia, including Kazakhstan, Kyrgyzstan, Mongolia, Tajikistan, Turkmenistan and Uzbekistan. Between 1990 and 2000, the age-standardized cirrhosis mortality rate rose by almost 60% in Kazakhstan and by 50% in Kyrgyzstan. Since 2000, the cirrhosis mortality rate in these countries has either stabilized or decreased. In 2010, Mongolia had the third-highest cirrhosis mortality in the world. This was largely a result of the high prevalence of hepatitis B and C viruses [40,41]. Actually, more than half of the liver cirrhosis cases in Central Asia were attributed to hepatitis B and C infection, 57% in 1990 and 54% in 2010 (Figure 2, Additional file 6: Table S6). Kyrgyzstan had the fifth-highest cirrhosis mortality rate globally in 2010; Uzbekistan and Turkmenistan ranked seventh and eighth, respectively. Similar increases in mortality also occurred in Pakistan and India between 1990 and 2000, although mortality from the disease appears to have gradually declined in the last decade. In 2010, there were an estimated 188,575 (109,748 to 303,989) liver cirrhosis deaths in India, accounting for almost one-fifth (18.3%) of the global liver cirrhosis death toll.

Between 1980 and 2010, age-standardized mortality rates from liver cirrhosis in China declined from 43.4 (26.6 to 68.1) per 100,000 to 16.2 (11.1 to 27.4) per 100,000, or by about two-thirds (Table 4; Figure 4). Declines in cirrhosis mortality have also been observed in Japan and South Korea. In Southeast Asia, cirrhosis mortality trends have been mixed, increasing in Indonesia, Sri Lanka and the Philippines, declining in Malaysia, Thailand, Singapore and Laos, and remaining unchanged in Cambodia, Myanmar and Vietnam (Table 4, Figure 4). The male-to-female ratio exceeded two in some countries of the region, namely the Republic of Korea and Japan, but was closer to one elsewhere in Asia.

In Egypt, treatment for schistosomiasis until the late 1960s led to widespread transmission of the hepatitis C virus [42]. Currently, Egypt has the highest prevalence of chronic hepatitis C infection in the world [43,44]. This is reflected in the very high liver cirrhosis mortality rates, particularly in males. In 2010, the age-standardized cirrhosis mortality rate in Egypt was the highest globally at 72.7 (57.2 to 87.2) per 100,000, despite a 25.9% reduction in mortality since 1980 (Table 4, Figure 4). During the same year, 18% of deaths in males between ages 45 to 54 years were due to liver cirrhosis.

In sub-Saharan Africa, liver cirrhosis deaths doubled between 1980 and 2010, from 53,000 (52,997: 27,116 to 97,496) to 103,000 (102,609: 53,005 to 185,330) in 2010 (Table 3). Cirrhosis mortality rates were about half as high in southern sub-Saharan Africa as compared to the central, eastern, and western regions of Africa, a pattern consistent with the distribution of hepatitis B and C infection [45–47].Cirrhosis mortality rates in the Central Africa Republic, Gabon, Malawi, Uganda and Cote d’Ivoire ranked in the highest tenth percentile globally in 2010. Liver cirrhosis was most commonly attributed to hepatitis B in Sub-Saharan Africa. Around 30% of cases were related to neither hepatitis infection nor alcohol intake (Figure 2, Additional file 6: Table S6).

## Discussion

Liver cirrhosis deaths worldwide have increased steadily over the past 30 years, exceeding one million in 2010, or approximately 2% of all deaths in that year. Somewhat paradoxically, there has been a concomitant decline in the age-standardized mortality rate, which decreased by 22% over the same period. This was due to population size and aging far outweighing the overall decline in cirrhosis mortality. Variations in mortality levels among regions and countries were predominantly driven by alcohol consumption levels, the type and quality of alcohol consumed, iatrogenic viral hepatitis C infection and viral hepatitis B infection.

In 2010, liver cirrhosis ranked as the 23rd cause of disease burden worldwide causing 31 million or 1.2% of global DALYs, with nearly equal proportions attributable to hepatitis B, hepatitis C and alcohol consumption [1]. Regionally, our findings suggest that liver cirrhosis is a particular health priority in Central Asia (ranked 9th among the leading causes of disease burden in 2010), Central Europe (rank 10), Eastern Europe (rank 11) and Central Latin America (rank 12). Alcohol related liver cirrhosis underlies the significant declines in cirrhosis burden in Europe over the past two decades, but also the substantial increases in Latin America. In Asia, more than half of the liver cirrhosis burden is attributable to hepatitis B and hepatitis C. Hepatitis B accounted for 44% and 42% of DALYs from liver cirrhosis estimated for East Asia and Central Asia, respectively, in 2010. In sub-Saharan Africa, the burden related to liver cirrhosis rose 57% from 1990 to 2010. In 2010, 34%, 18% and 17% of liver cirrhosis in the region was attributable to hepatitis B, alcohol intake and hepatitis C, respectively [1] (Figure 2, Additional file 6: Table S6).

**Figure 6:**
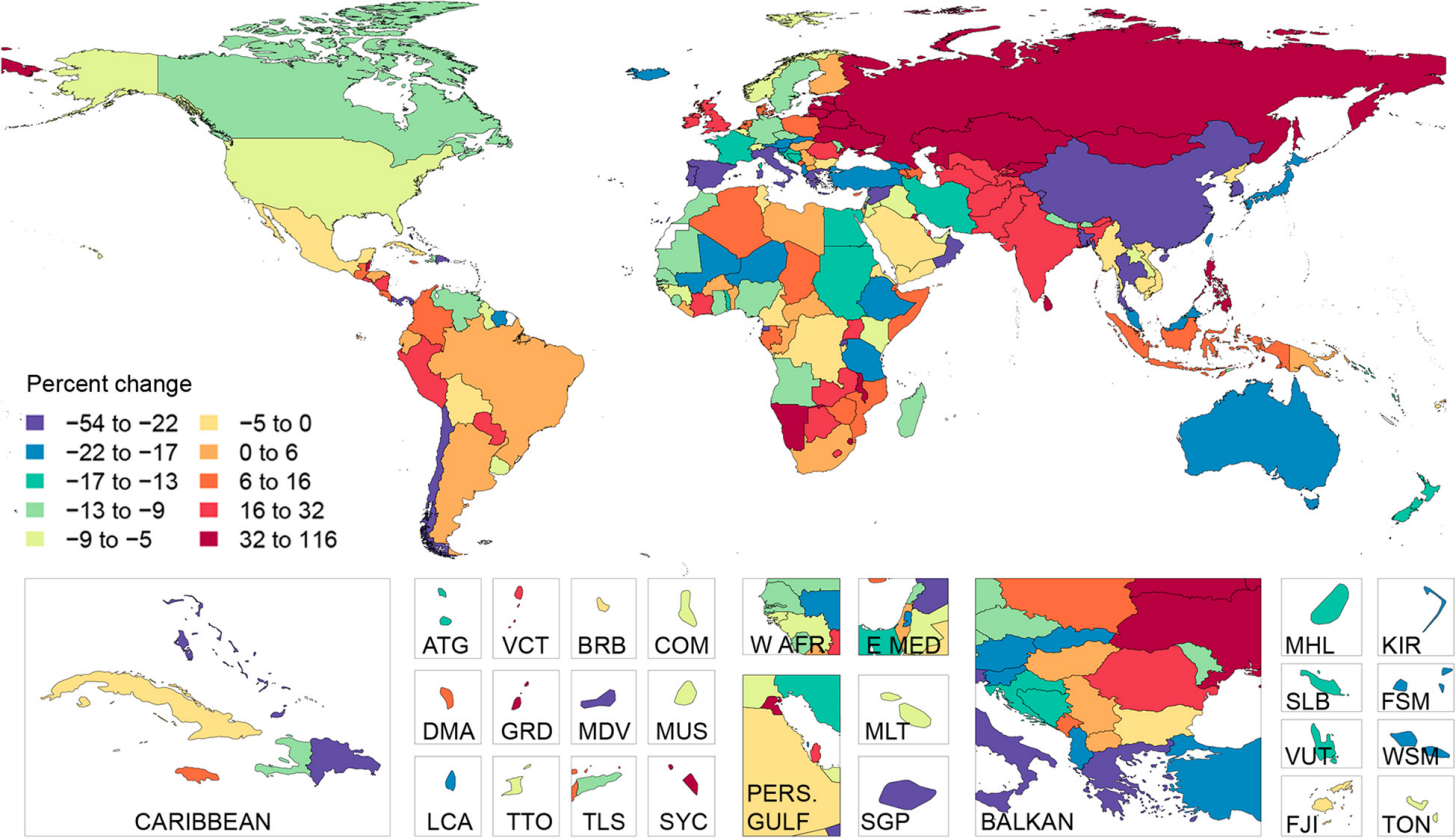
**Percent change in age-adjusted liver cirrhosis mortality between 1990 and 2000.** ATG(Antigua and Barbuda), BRB(Barbados), COM(Comoros), DMA(Dominica), E Med(East Mediterranean), FJI(Fiji), FSM(Micronesia, Federated States of), GRD(Grenada), KIR(Kiribati), LCA(Saint Lucia), MDV(Maldives), MHL(Marshall Islands), MLT(Malta), MUS(Mauritius), PERS GULF(Persian Gulf), SGP(Singapore), SLB(Solomon Islands), SYC(Seychelles), TLS(Timor-Leste), TON(Tonga), TTO(Trinidad and Tobago), VCT(Saint Vincent and the Grenadines), VUT(Vanuatu), W AFR(West Africa), WSM(Samoa).

In Eastern Europe, in the late 1980s and early 1990s, marked increases in liver cirrhosis mortality rates coincided with the dissolution of the Union of Soviet Socialist Republics (Figure 6). During this period, a dramatic increase in alcohol consumption, much of which was of poor quality and hepatotoxic, followed the removal of restrictions on the alcohol trade, particularly in Russia and Ukraine [5,48–50]. While alcohol generally requires several years to induce fibrosis in the liver, the comparatively short lag between national alcohol consumption levels and changes in cirrhosis mortality is probably related to the effects of alcohol on patients with subclinical compensated liver cirrhosis [4,38,51]. More recently, mortality has stabilized or even declined modestly in the region, although liver cirrhosis death rates remain alarmingly high, specifically in Moldova and Hungary. This may be largely attributable to widespread consumption of highly hepatotoxic home-brewed fruit-based alcoholic beverages in these countries [5,36–39,52–54].

The dramatic transformation in cirrhosis mortality accompanying the dissolution of the Soviet Union was also evident in Central Asia in Turkmenistan, Uzbekistan and Kyrgyzstan. With minimal alcohol intake in all three countries due to predominantly Muslim populations [25], liver cirrhosis is largely attributable to prevalent chronic viral hepatitis infections (Figure 2, Additional file 6: Table S6). In neighboring Mongolia, 99% of patients with liver cirrhosis had viral hepatitis, and at least 20% had dual hepatitis infection [41]. Possible factors underlying this high prevalence of hepatitis include unsafe surgical and dental procedures, inappropriate disposal of medical wastes, late introduction of blood screening, and mass vaccination for smallpox in the early decades of the 20th century using contaminated syringes [40,41,55].

South Asia, particularly India, is another region where priority attention to improve prevention and control of liver cirrhosis risk factors is needed, with almost one-fifth (18.3%) of global liver cirrhosis deaths in 2010 occurring in India alone. Cirrhosis mortality has been steadily increasing in India since 1980, as has alcohol consumption, prevalence of hepatitis B and C and diabetes (a major risk factor for nonalcoholic fatty liver disease (NAFLD)) [34]. This contrasts with reductions in liver cirrhosis mortality in China. With 93 million hepatitis B carriers, perinatally acquired hepatitis B is the leading cause of adult liver cirrhosis in China [42,56,57]. In 2005, a national hepatitis B control plan was introduced with the goal of reducing HBsAg seroprevalence to less than 1% in five-year-old children by 2010 [7]. Given that the effects of this national health program would be expected to appear only after several years, and possibly decades, in the future, it is unclear what factors are contributing to declining cirrhosis mortality in China. This may in part be related to a reduction in hepatitis B prevalence from 9.8% in 1992 to 7.2% in 2006 [57,58]. The decline in schistosomiasis secondary to introduction of praziquantel and the implementation of strict control programs may have also played a role in declining cirrhosis mortality in China [59,60]. Part of the decline in China may also be an artifact of the incorrect assignment of liver cirrhosis as an underlying cause of death for deaths actually due to schistosomiasis.

The decline in liver cirrhosis mortality in Western Europe, particularly in Spain, France and Italy, is undoubtedly due to a steady decrease in alcohol consumption, accompanied by an improvement in the quality of wine and restrictions on home-brewed alcoholic beverage making [5,36–39]. In contrast, the alarming increase in cirrhosis mortality in the UK recently prompted a national call by the Chief Medical Officer for prioritizing the prevention, identification and treatment of liver disease in the UK [61]. Dunbar *et al*. attributed this trend to a birth cohort effect [62], yet decomposition analysis of deaths in the UK show that such an effect would explain only about one-third of the increase in deaths over the period between 1990 and 2010 (Additional file 5: Table S5). A real increase in the age-standardized liver cirrhosis mortality rate in the UK is apparent, and was likely driven by an increase in alcohol consumption and a shift to a higher proportion of alcohol intake in the form of spirits and binge drinking outside of meals [25,63,64]. These two behaviors have been shown to increase the risk of developing liver cirrhosis in comparison with other alcohol types and drinking patterns [65–67]. Rising hepatitis C prevalence and a growing obesity epidemic in the UK have likely also played a role in increasing the liver cirrhosis burden over the past two decades [34,68,69].

Heavy alcohol consumption is likely to be the main cause of liver cirrhosis in most parts of Latin America [20,70–72] (Figure 2, Additional file 6: Table S6). However, large declines in cirrhosis mortality in Mexico and Chile were observed without significant changes in alcohol consumption. Furthermore, significant changes in alcohol consumption do not completely account for the downward trends in death rates in most countries of Central Latin America. Changing patterns of alcohol consumption with meals, and changes in the type of alcohol consumed may explain such trends, but little is known about the extent of these possible trends in Latin American societies. The prevalence of hepatitis B and C is generally low in these regions [43,69,73]. A role for treatment is less likely; case fatality rates in Latin America have either remained unchanged or may have increased between 1990 and 2010 (estimated from analysis of hospital data) [2]. Other studies have suggested an increasingly significant impact of NAFLD [71]; however, this is difficult to quantify based on existing data. There is an urgent need for further research to shed more light on these important changes in disease burden in the region.

We estimated more than 100,000 deaths in sub-Saharan Africa from liver cirrhosis in 2010, more than half of which were attributed to endemic hepatitis B and C (Figure 2, Additional file 6: Table S6). In 1991, the WHO undertook a global effort to incorporate hepatitis B vaccination into routine national immunization schedules. By 2007, 65% of African member states had included extended hepatitis B vaccination in their immunization schedule [73,74]. In comparison, hepatitis C, the other major driver of liver cirrhosis in Africa, is more difficult to control. Major modes of transmission were related to inadequate blood screening and iatrogenic medical causes [47,75–77]. Notably, in Uganda and Gabon, alcohol was a more predominant factor in comparison to other neighboring countries. Cirrhosis mortality rates in both these countries ranked among the highest tenth percentile globally in 2010. The general trend of liver cirrhosis mortality has stabilized in Sub-Saharan Africa recently; the country-level trends were more heterogeneous. These mortality estimates were largely based on verbal autopsy data, given the general lack of publicly available vital registration data in the region. Performance of verbal autopsy is suboptimal in accurately capturing the underlying cause of death. In the Population Health Metrics Research Consortium (PMHRC) study, Lozano *et al*. [78] demonstrated that physician-certified verbal autopsy accurately assigned liver cirrhosis as a cause of death in only 46% of cases. Also, Yang *et al*. [79] found considerable misclassification of viral hepatitis and chronic liver diseases in verbal autopsy data. Combining those entities, nevertheless, improved sensitivity of accurately detecting deaths due to liver diseases [79]. For a more complete estimation of the mortality envelope of liver diseases in sub-Saharan Africa, we included Table 5 that summarizes mortality rates and deaths due to liver cirrhosis, liver cancer, and acute hepatitis in the Sub-Saharan Africa regions. In 2010, liver disease was an underlying cause of death in 186,373 deaths in Sub-Saharan Africa. The temporal trend of liver diseases in this region mirrored those of liver cirrhosis discussed earlier, increasing number of deaths and down trending mortality rates.

**Table 5:** **Liver cirrhosis, liver cancer, and acute hepatitis deaths and mortality rates in Sub-Saharan Africa in years 1990 and 2010**

**Region**	**Liver cirrhosis**		**Liver cancer**		**Acute hepatitis**	
	**1990**	**2010**	**1990**	**2010**	**1990**	**2010**
**Sub-Saharan Africa, Central**	6,588(12.3)	11,549(12)	2,395(4.5)	3,912(4)	1,966(3.7)	2,539(2.6)
**Sub-Saharan Africa, East**	27,613(13.3)	40,219(11.3)	7,458(3.6)	12,889(3.6)	10,296(5)	15,986(4.5)
**Sub-Saharan Africa, Southern**	3,397(6.5)	6,227(8.8)	2,524(4.8)	2,493(3.5)	794(1.5)	829(1.2)
**Sub-Saharan Africa, West**	26,736(13.3)	44,892(13.4)	20,024(10)	25,705(7.7)	18,984(9.4)	19,133(5.7)
**Sub-Saharan Africa**	64,334(12.5)	102,887(12)	32,401(6.3)	44,999(5.2)	32,040(6.2)	38,487(4.5)

In parentheses, mortality rate (per 100,000).

A particularly alarming finding of our study pertains to iatrogenic causes, particularly the reuse of syringes in health facilities, in the transmission and creation of large population reservoirs of hepatitis C in low-income countries. This was particularly true of Egypt and Central Africa following mass injecting campaigns for parenteral therapy for schistosomiasis and trypanosomiasis, respectively [47,80–82], as well as in Pakistan, Ethiopia and several other countries where the reuse of syringes in health facilities has been common practice [75,76,81].

We conclude that the main drivers of mortality and disease burden from liver cirrhosis over the past two decades are likely to have been the substantial, yet heterogeneous changes in the key risk factors for the disease. At most, a minor role might be attributable to improved treatment of complications of liver cirrhosis. Roberts *et al*. [21] demonstrated that mortality rates did not improve after admission for liver cirrhosis in the UK between 1968 and 1999. While liver transplant has been shown to improve survival [83], the number of transplanted patients is not sufficient to account for much of the decline in liver cirrhosis mortality. In the US, for example, approximately 6,000 livers are transplanted annually [84]; this represents only 5% of patients with decompensated liver cirrhosis [1,26]. Globally, the case fatality rate of patients with decompensated liver cirrhosis (estimated from an analysis of hospital data) remained unchanged, at about 20% (95% uncertainty interval: 15 to 26) in 1990 and 22% (16 to 28) in 2010, even in more developed countries [1,2,26].

Our analysis has several limitations, five of which pertain to the particular case of liver cirrhosis mortality. First, incomplete cause of death data and reliance on verbal autopsy data, particularly in countries in sub-Saharan Africa, greatly increase the uncertainty of our findings. We have tried to quantify this uncertainty, but it means that our estimates of mortality rates and trends in these countries need to be viewed cautiously. Second, cause of death data may contain significant diagnostic misclassification, even when medically certified [85–87]. Extensive misclassification of deaths in hospitals by physicians, especially in developing countries, has been widely documented [88–90]. This is more pronounced in verbal autopsy data, particularly in the case of sub-Saharan Africa as discussed above. A brief discussion of liver cancer and acute hepatitis deaths in sub-Saharan Africa is included to allow a better appreciation of the burden related to the main drivers of fatal liver disease in the region, viral hepatitis, and a more complete estimation of the mortality envelope of liver diseases in sub-Saharan Africa. Third, while we believe that our redistribution algorithms have improved comparability across ICD revisions and reduced the impact of garbage codes, there is still uncertainty surrounding the redistribution algorithms employed [13]. Fourth, a broad definition for liver cirrhosis was used for reasons listed elsewhere in this manuscript, which may have inflated our mortality estimates. Fifth, we have excluded hepatocellular carcinoma from our definition, as discussed under the section ‘case definition of liver cirrhosis’, which could underestimate true liver cirrhosis mortality.

## Conclusions

We estimate that mortality from liver cirrhosis accounts for a growing and substantial disease burden worldwide each year, causing more than one million deaths in 2010. Treatment of this disease is expensive and is largely inaccessible in most parts of the world. In addition, with the exception of liver transplantation, treatment for cirrhosis has been shown to, at most, minimally improve long-term survival in patients with decompensated liver cirrhosis [21,91,92]. By contrast, preventive measures such as screening transfused blood for viral hepatitis, employing appropriate medical hygiene in health facilities, introducing hepatitis B vaccination programs, imposing alcohol trade restrictions and raising taxes on alcohol, implementing health promotion and education programs to reduce alcohol consumption and harmful alcohol use are relatively inexpensive and cost-effective [3–5,43,93,94]. Our study strongly suggests that a more purposeful and widespread application of such measures in national health policies is urgently required, particularly in poor countries, to address this growing and largely unappreciated global health concern.

### Endnotes

^a^Mortality rates were standardized to the WHO standard population for ages 0 to 100 years [95].

^b^See Additional file 10: Table S7 for a regional categorization of countries used in GBD 2010.

## Additional files

Additional file 1: Table S1. Death by icd. Fraction of total deaths cirrhosis by ICD code. Additional file 2: Table S2. Data sources. Individual data sources. Additional file 3: Table S3. GC redistribution. Garbage code redistribution onto liver cirrhosis. Additional file 4: Table S4 a. Male submodels. Male liver cirrhosis ensemble model component submodels with weights. **Table S4b**. Female submodels. Female liver cirrhosis ensemble model component submodels with weights. Additional file 5: Table S5. Decomposition. Decomposition of liver cirrhosis deaths by population growth, population aging, and death rates. Additional file 6: Table S6. PAFs by region. Population attributable fractions for liver cirrhosis risk factors by region in 1990 and 2010. Additional file 7: Figure S1. Map ASdr sexratio2010. A global map of male to female liver cirrhosis mortality sex ratios. The figure title and captions are as follows: Male to female mortality ratio in 2010. ATG(Antigua and Barbuda), BRB(Barbados), COM(Comoros), DMA(Dominica), E Med(East Mediterranean), FJI(Fiji), FSM(Micronesia, Federated States of), GRD(Grenada), KIR(Kiribati), LCA(Saint Lucia), MDV(Maldives), MHL(Marshall Islands), MLT(Malta), MUS(Mauritius), PERS GULF(Persian Gulf), SGP(Singapore), SLB(Solomon Islands), SYC(Seychelles), TLS(Timor-Leste), TON(Tonga), TTO(Trinidad and Tobago), VCT(Saint Vincent and the Grenadines), VUT(Vanuatu), W AFR(West Africa), WSM(Samoa). Additional file 8: Figure S2. Map ASdr male 2010. A global map of male liver cirrhosis mortality in 2010 at the country level. The figure title and captions are as follows: Age-adjusted liver cirrhosis mortality (per 100,000) for males in 2010. ATG(Antigua and Barbuda), BRB(Barbados), COM(Comoros), DMA(Dominica), E Med(East Mediterranean), FJI(Fiji), FSM(Micronesia, Federated States of), GRD(Grenada), KIR(Kiribati), LCA(Saint Lucia), MDV(Maldives), MHL(Marshall Islands), MLT(Malta), MUS(Mauritius), PERS GULF(Persian Gulf), SGP(Singapore), SLB(Solomon Islands), SYC(Seychelles), TLS(Timor-Leste), TON(Tonga), TTO(Trinidad and Tobago), VCT(Saint Vincent and the Grenadines), VUT(Vanuatu), W AFR(West Africa), WSM(Samoa). Additional file 9: Figure S3. Map ASdr female 2010. A global map of female 1liver cirrhosis mortality in 2010 at the country level. The figure title and captions are as follows: Age-adjusted liver cirrhosis mortality (per 100,000) for females in 2010. ATG(Antigua and Barbuda), BRB(Barbados), COM(Comoros), DMA(Dominica), E Med(East Mediterranean), FJI(Fiji), FSM(Micronesia, Federated States of), GRD(Grenada), KIR(Kiribati), LCA(Saint Lucia), MDV(Maldives), MHL(Marshall Islands), MLT(Malta), MUS(Mauritius), PERS GULF(Persian Gulf), SGP(Singapore), SLB(Solomon Islands), SYC(Seychelles), TLS(Timor-Leste), TON(Tonga), TTO(Trinidad and Tobago), VCT(Saint Vincent and the Grenadines), VUT(Vanuatu), W AFR(West Africa), WSM(Samoa). Additional file 10: Table S7. Region country. Regional categorization of countries in the GBD 2010 study.

## Abbreviations

CODEm: cause of death ensemble modeling
DALYs: disability adjusted life years
GBD: global burden of disease
HBsAg: hepatitis B surface antigen
HBV: hepatitis B virus
HCC: hepatocellular carcinoma
HCV: hepatitis C virus
ICD: International Classification of Diseases
NAFLD: non-alcoholic fatty liver disease
PAF: population attributable fractions
WHO: World Health Organization
