# Supplementary figures and images for: Liver cirrhosis mortality in 187 countries between 1980 and 2010: a systematic analysis
Source: BMC Med. 2014 Sep 18;12:145. doi: 10.1186/s12916-014-0145-y (PMC4169640; doi:10.1186/s12916-014-0145-y)

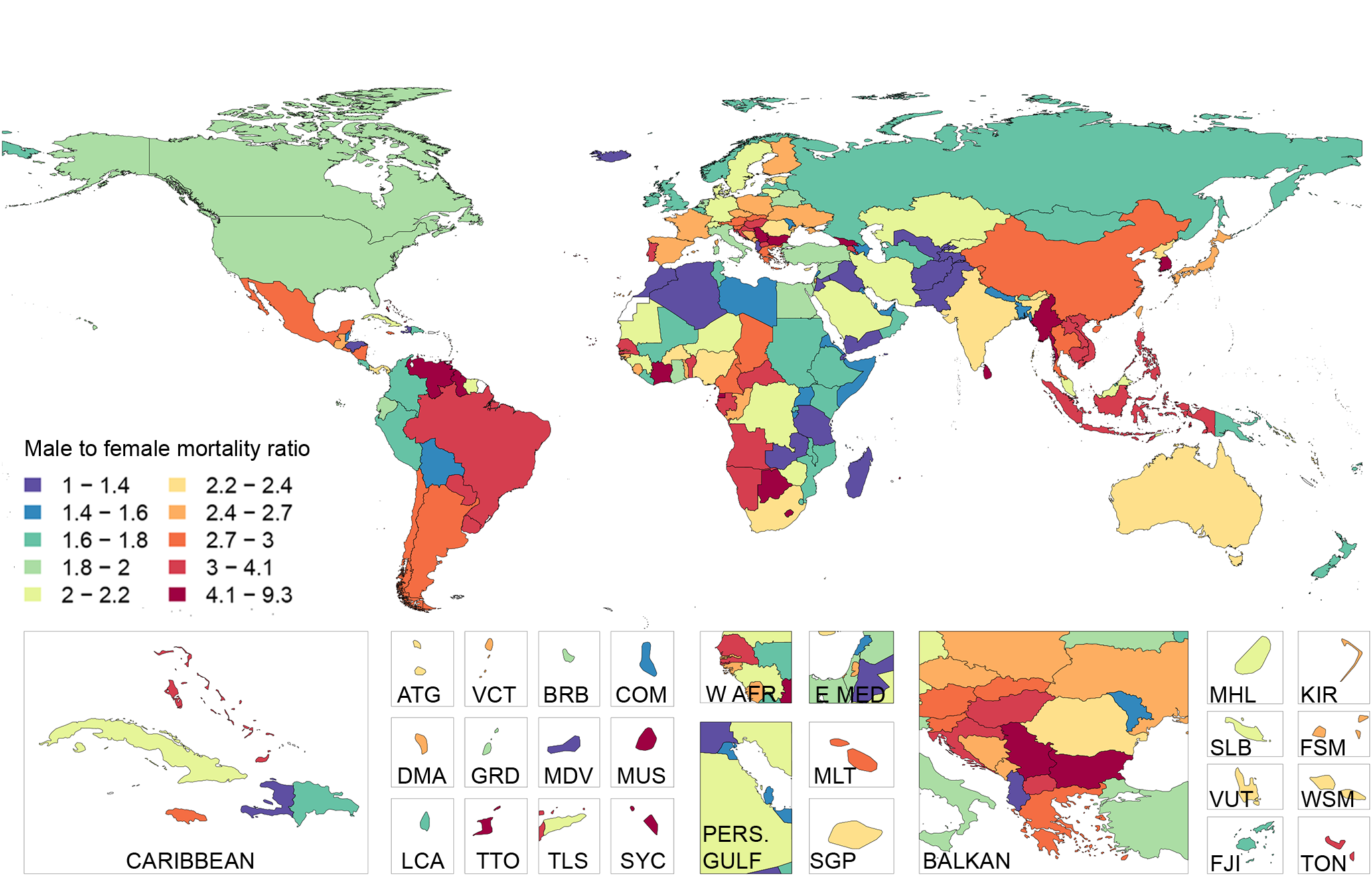

Supplement: Additional file 7: Figure S1. — Map ASdr sexratio2010. A global map of male to female liver cirrhosis mortality sex ratios. The figure title and captions are as follows: Male to female mortality ratio in 2010. ATG(Antigua and Barbuda), BRB(Barbados), COM(Comoros), DMA(Dominica), E Med(East Mediterranean), FJI(Fiji), FSM(Micronesia, Federated States of), GRD(Grenada), KIR(Kiribati), LCA(Saint Lucia), MDV(Maldives), MHL(Marshall Islands), MLT(Malta), MUS(Mauritius), PERS GULF(Persian Gulf), SGP(Singapore), SLB(Solomon Islands), SYC(Seychelles), TLS(Timor-Leste), TON(Tonga), TTO(Trinidad and Tobago), VCT(Saint Vincent and the Grenadines), VUT(Vanuatu), W AFR(West Africa), WSM(Samoa). [file 12916_2014_145_MOESM7_ESM.tiff]

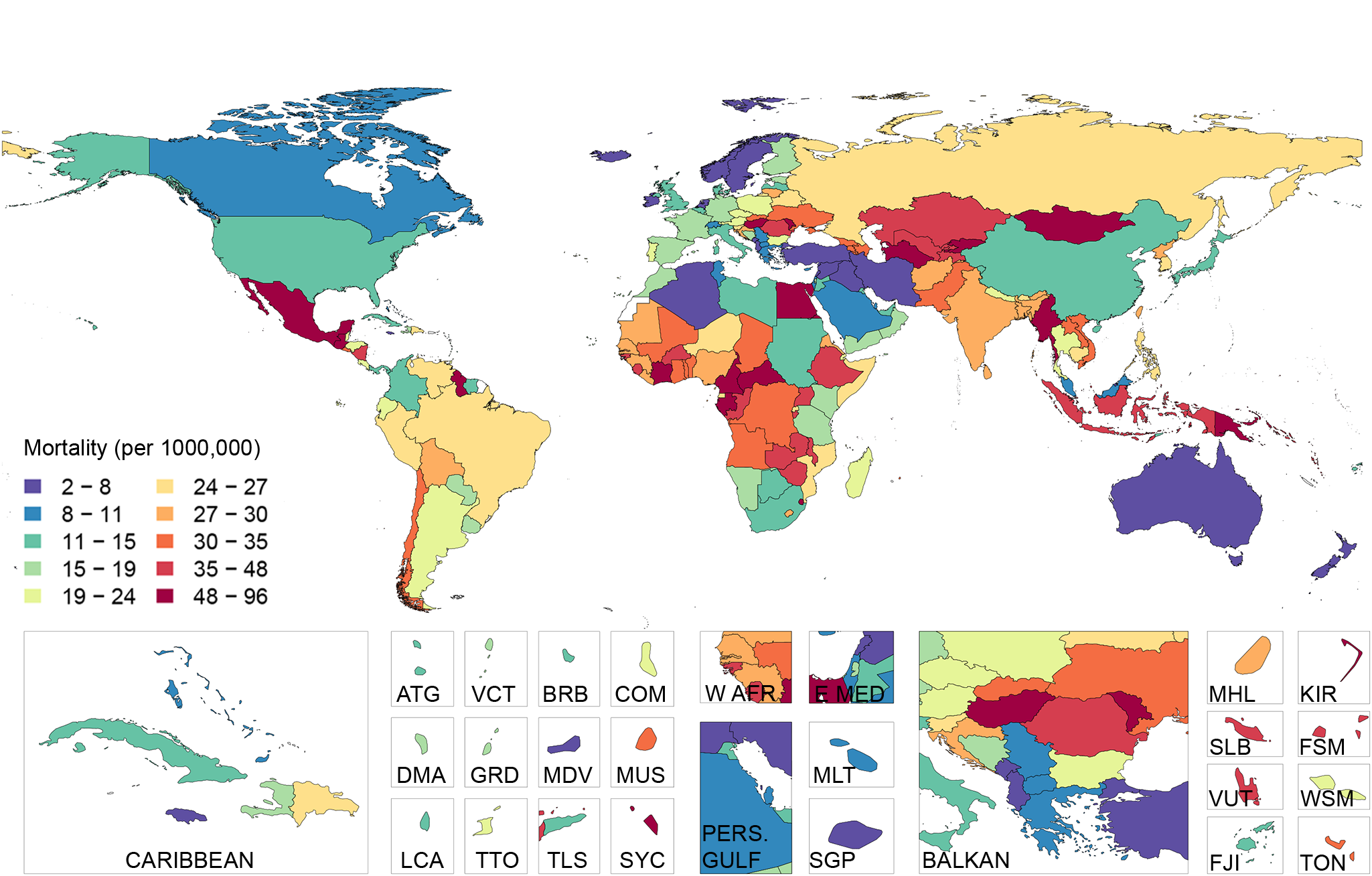

Supplement: Additional file 8: Figure S2. — Map ASdr male 2010. A global map of male liver cirrhosis mortality in 2010 at the country level. The figure title and captions are as follows: Age-adjusted liver cirrhosis mortality (per 100,000) for males in 2010. ATG(Antigua and Barbuda), BRB(Barbados), COM(Comoros), DMA(Dominica), E Med(East Mediterranean), FJI(Fiji), FSM(Micronesia, Federated States of), GRD(Grenada), KIR(Kiribati), LCA(Saint Lucia), MDV(Maldives), MHL(Marshall Islands), MLT(Malta), MUS(Mauritius), PERS GULF(Persian Gulf), SGP(Singapore), SLB(Solomon Islands), SYC(Seychelles), TLS(Timor-Leste), TON(Tonga), TTO(Trinidad and Tobago), VCT(Saint Vincent and the Grenadines), VUT(Vanuatu), W AFR(West Africa), WSM(Samoa). [file 12916_2014_145_MOESM8_ESM.tiff]

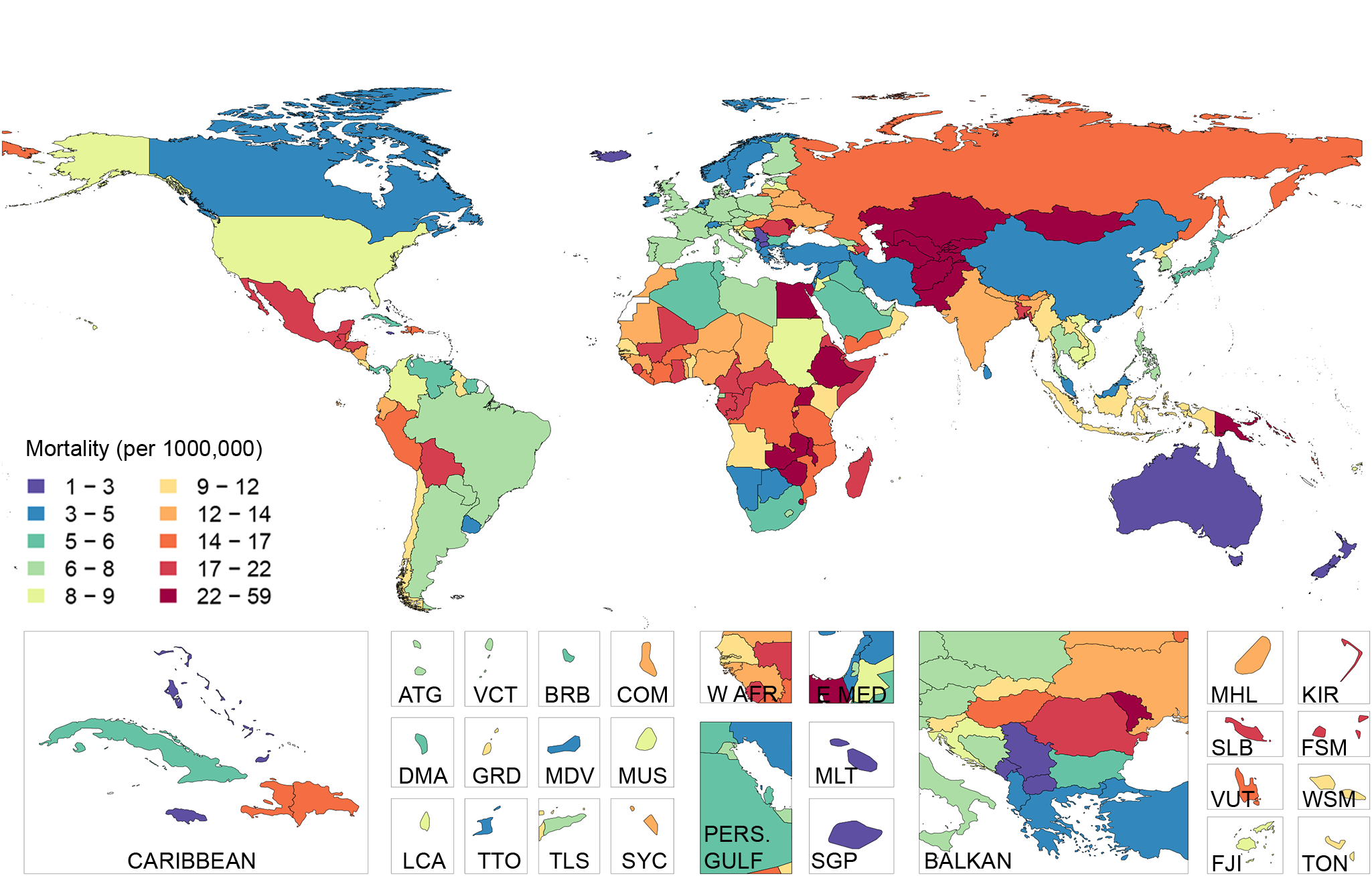

Supplement: Additional file 9: Figure S3. — Map ASdr female 2010. A global map of female 1liver cirrhosis mortality in 2010 at the country level. The figure title and captions are as follows: Age-adjusted liver cirrhosis mortality (per 100,000) for females in 2010. ATG(Antigua and Barbuda), BRB(Barbados), COM(Comoros), DMA(Dominica), E Med(East Mediterranean), FJI(Fiji), FSM(Micronesia, Federated States of), GRD(Grenada), KIR(Kiribati), LCA(Saint Lucia), MDV(Maldives), MHL(Marshall Islands), MLT(Malta), MUS(Mauritius), PERS GULF(Persian Gulf), SGP(Singapore), SLB(Solomon Islands), SYC(Seychelles), TLS(Timor-Leste), TON(Tonga), TTO(Trinidad and Tobago), VCT(Saint Vincent and the Grenadines), VUT(Vanuatu), W AFR(West Africa), WSM(Samoa). [file 12916_2014_145_MOESM9_ESM.tiff]
